# Diagnostic and therapeutic practices in adult chronic nonbacterial osteomyelitis (CNO)

**DOI:** 10.1186/s13023-023-02831-1

**Published:** 2023-07-21

**Authors:** A. T. Leerling, G. Clunie, E. Koutrouba, O. M. Dekkers, N. M. Appelman-Dijkstra, E. M. Winter

**Affiliations:** 1 Department of Internal Medicine, Division of Endocrinology, Leiden University Medical Center, Leiden, the Netherlands; 2 Center for Bone Quality, Leiden University Medical Center, Leiden, the Netherlands; 3 Department of Clinical Epidemiology, Leiden University Medical Center, Leiden, the Netherlands; 4 Department of Rheumatology, Cambridge University Hospitals NHS Foundation Trust, Cambridge, UK

**Keywords:** Chronic nonbacterial osteomyelitis, SAPHO, Sternocostoclavicular hyperostosis, Pustulotic arthro-osteitis, Diagnostics, Treatment, Survey

## Abstract

**Background:**

Chronic nonbacterial osteomyelitis (CNO) is a rare, and impactful auto-inflammatory bone disease occurring in children and adults. Clinical care for CNO is challenging, as the condition lacks validated classification criteria and evidence-based therapies. This study aimed to map the current diagnostic and therapeutic practices for CNO in adults, as a first step towards a standardized disease definition and future consensus treatment plans.

**Methods:**

A primary survey was spread among global rheumatological/bone networks and 57 experts as identified from literature (May 2022), covering terminology, diagnostic tools (clinical, radiological, biochemical) and treatment steps. A secondary survey (sent to primary survey responders in August 2022) further queried key diagnostic features, treatment motivations, disease activity and treatment response monitoring.

**Results:**

36 and 23 physicians completed the primary and secondary survey respectively. Diagnosis was mainly based on individual physician assessment, in which the combination of chronic relapsing-remitting bone pain with radiologically-proven osteitis/osteomyelitis, sclerosis, hyperostosis and increased isotope uptake on bone scintigraphy were reported indicative of CNO. Physicians appeared more likely to refer to the condition as synovitis, acne, pustulosis, hyperostosis, osteitis (SAPHO) syndrome in the presence of joint and skin pathology. MRI was most frequently performed, and the preferred diagnostic test for 47%. X-rays were second-most frequently used, although considered least informative of all available tools. Typical imaging features reported were hyperostosis, osteitis, osteosclerosis, bone marrow edema, while degeneration, soft tissue calcification, and ankylosis were not regarded characteristic. Inflammation markers and bone markers were generally regarded unhelpful for diagnostic and monitoring purposes and physicians infrequently performed bone biopsies. Management strategies diverged, including indications for treatment, response monitoring and declaration of remission. Step-1 treatment consisted of non-steroidal anti-inflammatory drugs/COX-2 inhibitors (83%). Common step 2–3 treatments were pamidronate, methotrexate, and TNF-a-inhibition (anti-TNFα), the latter two regarded especially convenient to co-target extra-skeletal inflammation in SAPHO syndrome. Overall pamidronate and anti-TNFα and were considered the most effective treatments.

**Conclusions:**

Following from our survey data, adult CNO is a broad and insufficiently characterized disease spectrum, including extra-osseous features. MRI is the favoured imaging diagnostic, and management strategies vary significantly. Overall, pamidronate and anti-TNFα are regarded most successful. The results lay out current practices for adult CNO, which may serve as backbone for a future consensus clinical guideline.

**Supplementary Information:**

The online version contains supplementary material available at 10.1186/s13023-023-02831-1.

## Introduction

Chronic nonbacterial osteomyelitis (CNO, ORPHA: 324,964) is a rare disease spectrum marked by auto-inflammatory bone lesions and locally elevated bone turnover. Patients may present with inflammatory bone pain, swelling and functional impairment, which profoundly impacts quality of life [1, 2]. Over time, persistent bone inflammation may lead to irreversible structural tissue damage, characterized by sclerosis, erosions, new bone formation, and degenerative changes, emphasizing the need for timely diagnosis and intervention [3, 4]. Despite the increasing awareness for CNO in the past decades, clinical management remains challenging due to the absence of evidence-based treatments [5]. CNO demonstrates a broad clinical phenotype, encompassing both paediatric and adult presentations. In children, CNO is characterized by multiple alternating bone lesions that mostly affect the long bones and is usually referred to as chronic recurrent multifocal osteomyelitis (CRMO, shared ORPHA code with CNO: 324,964). Adult CNO comprises different subtypes, including sternocostoclavicular hyperostosis (SCCH, ORPHA: 178,311), indicating specific involvement of the anterior chest wall, and Synovitis, Acne, Pustulosis, Hyperostosis, Osteitis (SAPHO) syndrome, indicating more systemic involvement with joint and skin pathology (ORPHA: 793). Adults may also display a multifocal disease pattern that resembles paediatric CRMO [6–9]. At present, it is unknown to what degree the adult CNO-spectrum shares a common genetic or immunological base and should be addressed as a coherent entity, or whether subtypes differ in pathophysiology warranting different therapeutic approach. In any case, there are no validated classification criteria for the adult CNO spectrum as a whole, nor for its (presumed) subtypes. Proposed criteria for the CNO-spectrum were either derived from paediatric populations, or aim to frame the broad and heterogeneous entity of SAPHO syndrome [10–12] [9, 13].The unclear definition of adult CNO is problematic as it complicates research on diagnostic modalities, the evaluation of effectiveness of different treatments and disease monitoring tools. Importantly, randomized treatment trials are lacking for adult CNO, leading to clear variation in treatment regimens, monitoring tools and reported response rates between countries, centres, and medical disciplines [9]. Hence, there is an urgent need for a diagnostic and management guideline for adult CNO, including expert-based recommendations for as long as evidence-based ones are lacking. The objective of this study was to collect physician’s perspectives on the diagnosis and treatment of adult CNO, which may serve as backbone for a future consensus guideline.

## Materials & methods

A primary and secondary survey were designed by the Center for Bone Quality of the Leiden University Medical Center, the Netherlands, the Dutch CNO expert centre. The surveys were facilitated by the software of EUSurvey, an online survey-management system [14]. The initial focus of the survey was adults with sterile bone inflammation of the anterior chest wall (CNO/SCCH; ORPHA 178,311), considering its status as the most distinct and prevalent clinical expression of adult CNO [7, 9]. However, the survey invitation also incorporated the term SAPHO, as this name is more widely recognized in rheumatological networks and is closely linked to the disease concept of interest. Also, the survey specifically queried additional locations of bone lesions and extra-osseous features to evaluate the width of the disease concept as experienced by physicians, and test to what extent the initial CNO/SCCH-focus was supported. Together, the surveys covered topics of diagnostic features, nomenclature, diagnostic work-up and tools, treatment, and treatment response monitoring (**additional file 1** for full primary and secondary survey). Questions were designed as drop-down (“only one option possible”) or checkboxes (“check all that apply”), except for a small number of open text questions as indicated. The primary survey was developed as an open survey and included an early exit question to ensure that responders were care providers for adult CNO patients. Between May and August 2022, the primary survey was spread via 12 rheumatology, bone and rare disease networks (**additional file 2** for overview) and additionally to 57 individual experts as identified either within the Centre’s network, or as first or last authors of adult CNO publications in the preceding five years. For those approached personally, two reminders were sent after the initial invitation. A secondary survey, containing more in-depth questions was sent to primary survey responders late August 2022, with two reminders as appropriate. SPSS 25 (IBM Corp) was used for analyses. Data of multiple-choice questions are presented as frequency of counts or proportions. Likert scales for diagnostic features (*irrelevant/a bit relevant/relevant but not essential/quite essential/indispensable*) were converted into numerical scores (1, 2, 3, 4, 5 points respectively); medians and interquartile ranges (IQR) were calculated as indicated. The free text question on the diagnostic definition of CNO was analysed by generating response categories by two independent researchers (EK AND ATL; eventual categorization consensus-based). Numerical data are presented as mean ± standard deviation (SD) or median (IQR) for normal and non-normal data, as assessed by graphical inspection and Shapiro-Wilk test. Data are structured based on content; figures and tables indicate whether data items derived from primary or secondary survey.

## Results

### Characteristics of respondents

**Fig. 1:**
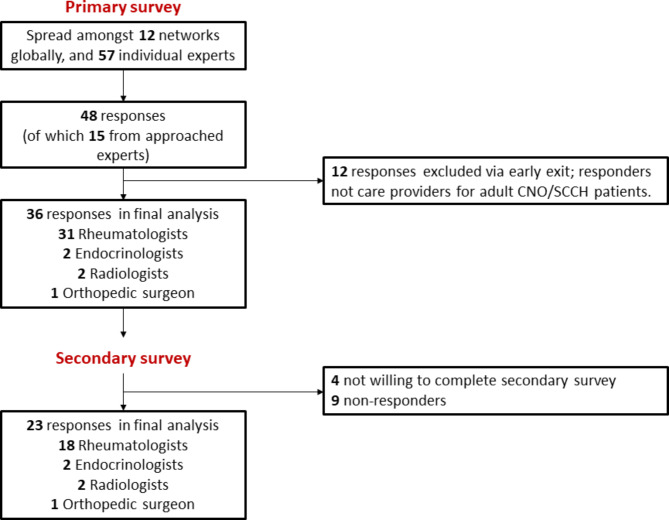
Overview of survey responders

**Fig. 2:**
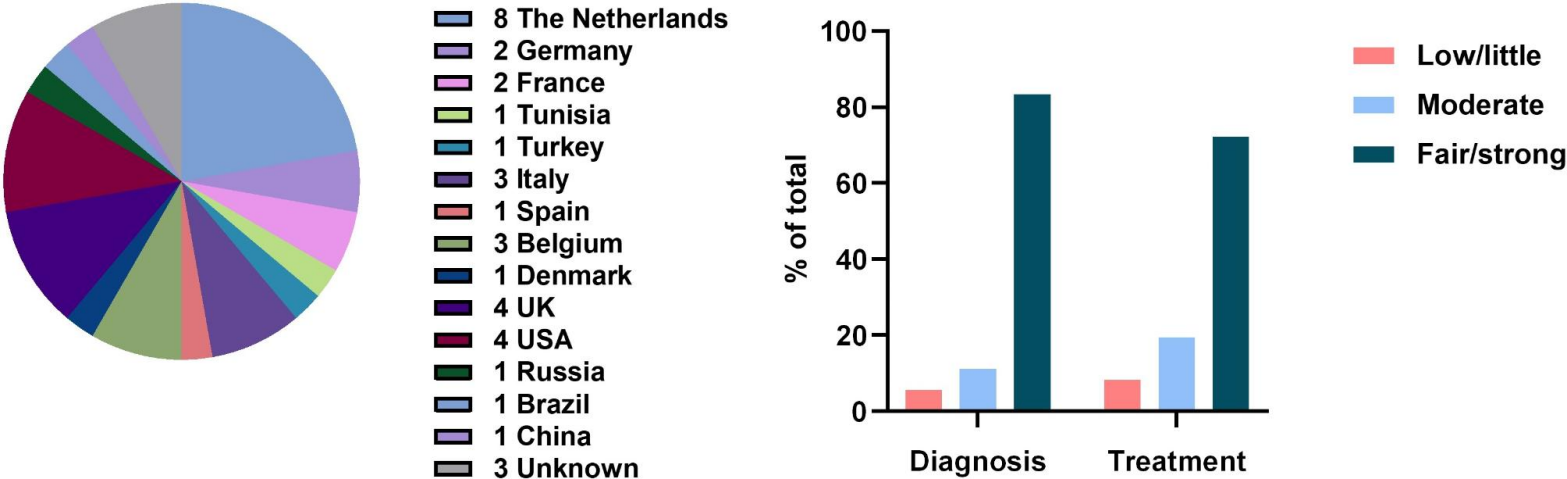
**2A (left)**: Country of residence of respondents (primary survey, n = 36) **2B (right)**: Level of confidence of physicians in diagnosing and treating adult CNO/SCCH (primary survey, n = 36)

48 physicians completed the primary survey, of whom 36, mainly rheumatologists, represented the target population of care providers for adult CNO/SCCH patients (Fig. 1). Of these, 23 completed the secondary survey. Responses mainly derived from Europe (64%), and also from North America (11%), South America (3%), Asia (6%) and Africa (3%) (Fig. 2A). Physicians were mostly based at university medical centres (86%), non-university medical centres (19%) and several practiced at private clinics (8%). Most physicians had > 10 or 5–10 years of clinical experience (78% and 14% respectively). Median number of patients referred for suspected CNO/SCCH was 5 per year (IQR 2–14). 64% stated to see 1–5 confirmed patients annually, 22% see 1–5 patients per month, and 14% see 1–5 patients per week. Physicians were mostly strongly or fairly confident in diagnosing and treating adult CNO/SCCH (83% and 72% respectively) (Fig. 2B).

### Nomenclature

**Fig. 3:**
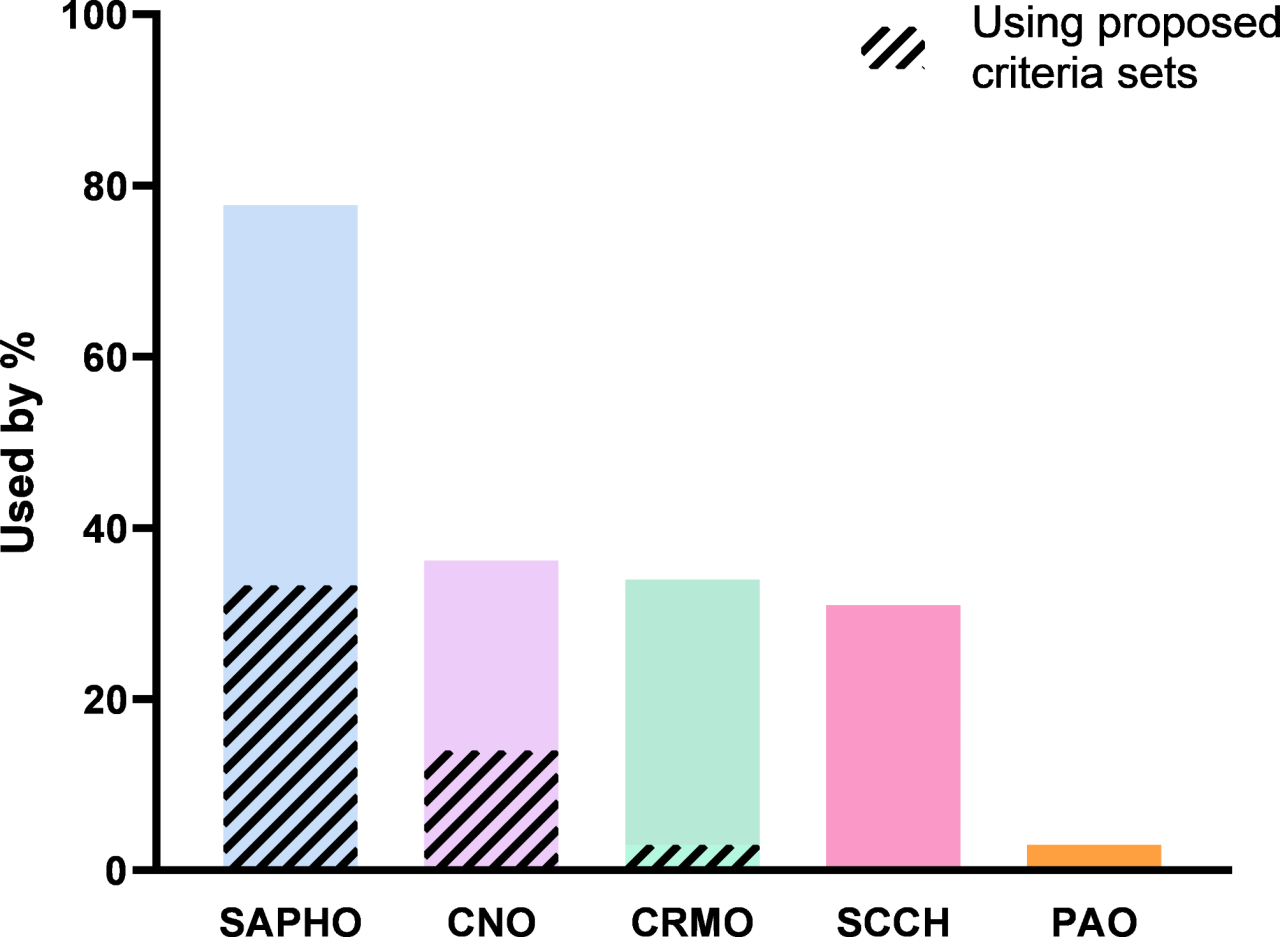
Labels used to describe CNO/SCCH (primary survey, n = 36). *Legend: Proposed criteria sets: Kahn 1994/2003 for SAPHO, Bristol 2016 or Jansson 2007 for CNO/CRMO (derived from paediatric cohorts).* See footnote for abbreviations 1

Given the variety of labels used to indicate the patient population of CNO/SCCH, the survey inquired about the use of these labels in clinical practice. 50% of physicians used one term consistently to describe adult CNO/SCCH, whereas 50% used multiple terms depending on the clinical picture. SAPHO was used most frequently (78%) (Fig. 3). CNO and CRMO were used by 36% and 33% respectively and SCCH was used by 31% (solely by physicians from The Netherlands, Belgium and Germany). Among physicians favouring ‘SAPHO’ and/or ‘CNO/CRMO’ as terms, a minority indicated to adhere to the criteria proposed for these labels, that is the criteria of Kahn 1994/2003 for SAPHO, and the criteria deriving from paediatric studies for CNO/CRMO (Bristol 2016 or Jansson 2007) [10–12, 15]. Pustulotic arthro-osteitis (PAO), a term that derives from dermatology and indicates bone inflammation in the context of pustulosis palmoplantaris (PPP), was used by a single physician.

### Clinical presentation & diagnostic features

**Fig. 4:**
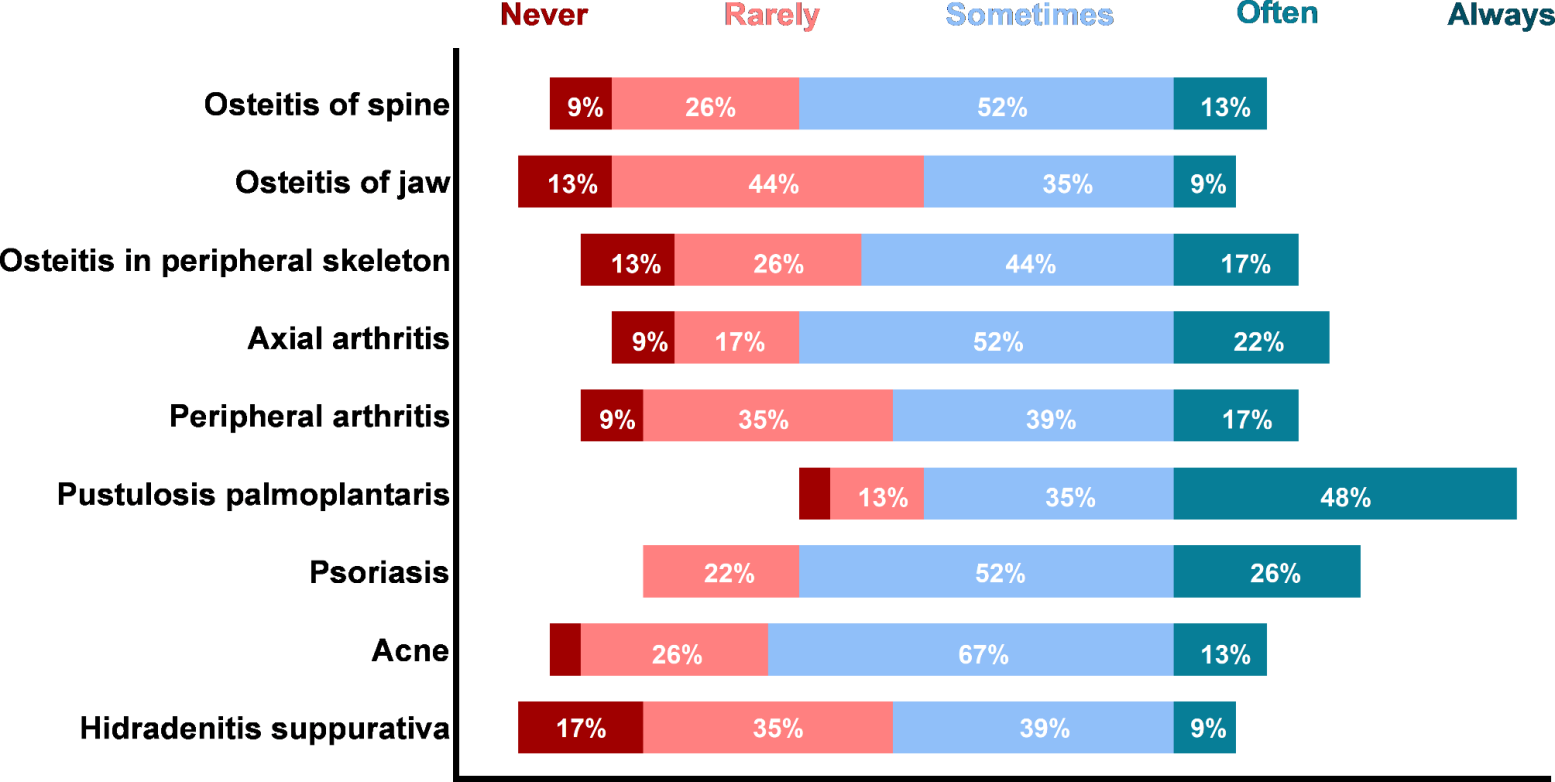
Presence of clinical features in adult CNO patients (secondary survey, n = 23)

The presence of clinical features as observed in adult CNO/SCCH (i.e. in adults with sterile bone inflammation of the anterior chest wall) is depicted in Fig. 4. Generally, many features are seen in “some” cases, reflecting a heterogeneous clinical presentation. Physicians reported different frequencies osteitis of the jaw, peripheral arthritis, and hidradenitis suppurativa; these were scored occurring as “never” (13%, 13%, 17%) almost as frequently as “often” (9%, 17%, 9%). Most consistently rated as prevalent features were PPP, followed by psoriasis, axial arthritis and peripheral and spinal osteitis. Other rare features that (single) physicians reported in an open field question are listed in **additional file 3.**

**Fig. 5:**
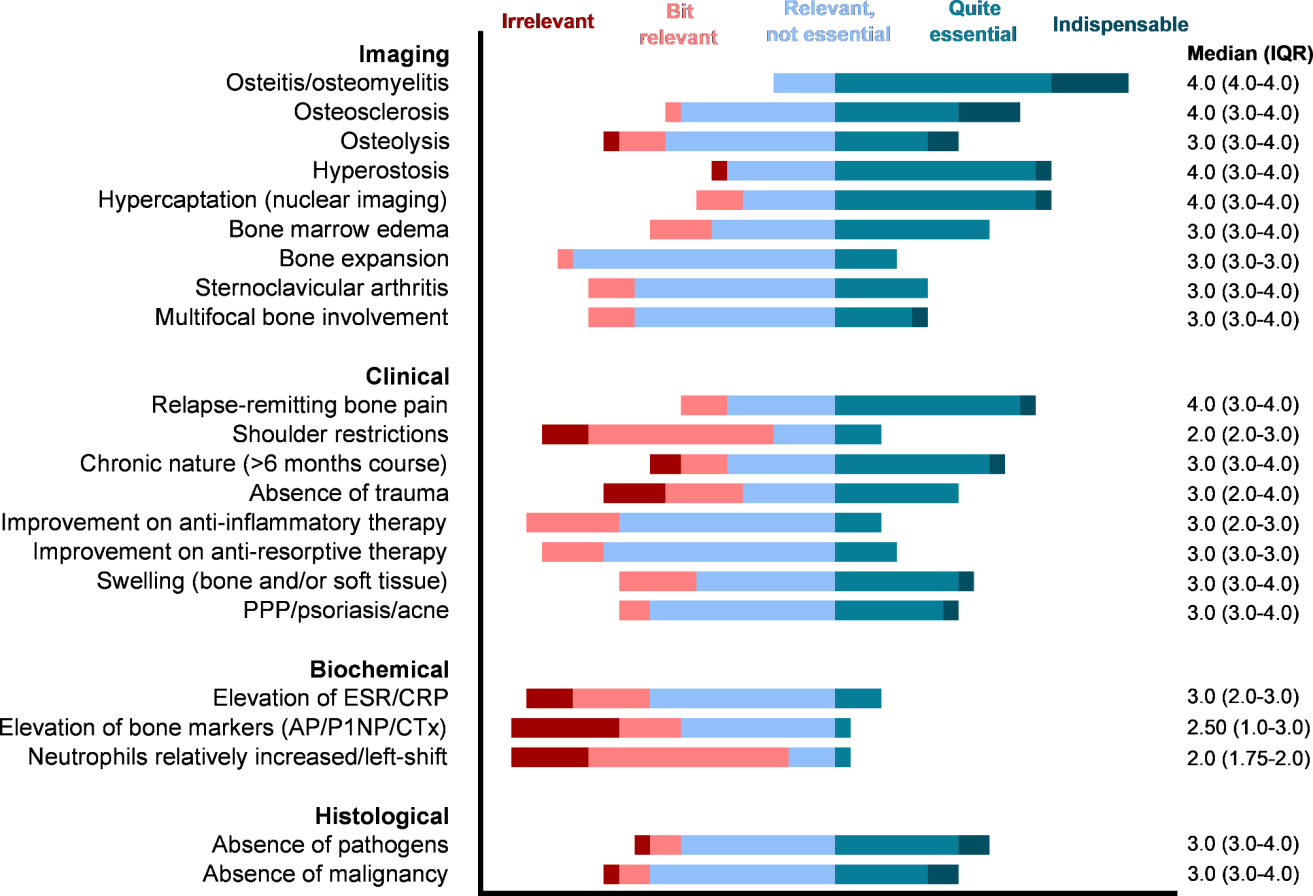
Relevance of individual clinical features to diagnose adult CNO/SCCH (5-point Likert scale: irrelevant, bit relevant, relevant but not essential, quite essential, indispensable; secondary survey, n = 23). See footnote for abbreviations^2^

To gain a deeper understanding of the physician’s conceptualization of CNO/SCCH, the survey included a follow-up question for physicians who indicated to *not* use diagnostic criteria sets to identify their patients (n = 25) (**additional file 3)**. A free-text diagnostic definition of adult CNO/SCCH was asked. Following categorization of the responses, the definition seemed to rely mostly on imaging. Imaging features alone (n = 7) or in conjunction with distinctive clinical features (n = 7) were leading in diagnosing adult CNO/SCCH. A minority of physicians (n = 3) emphasized the necessity of histological evidence to confirm the presence of sterile bone inflammation. Furthermore, 3 physicians indicated that diagnosis of adult CNO/SCCH requires the exclusion of other diagnoses such as axial spondylarthritis. Importantly, the majority of responses did not incorporate the anterior chest wall as a specifically typical localization. Instead, responses embodied the broader concept of “sterile bone inflammation, with or without accompanying extra-skeletal inflammatory manifestations”. Building on the free-text input of physicians and use of existing criteria sets, physicians were asked to score the *individual relevance* of specific features to diagnose adult CNO/SCCH (Fig. 5). Most positively rated were radiologically proven osteitis/osteomyelitis (median (IQR) 4.0 (4.0–4.0)), relapse-remitting bone pain, sclerosis, hyperostosis, and increased uptake on nuclear imaging (all 4.0 (3.0–4.0)). Shoulder movement restriction and biochemical parameters were generally rated less or irrelevant. The remainder of features were rated mostly neutral, as relevant but not essential for diagnosis.

### Diagnostic tools

**Fig. 6:**
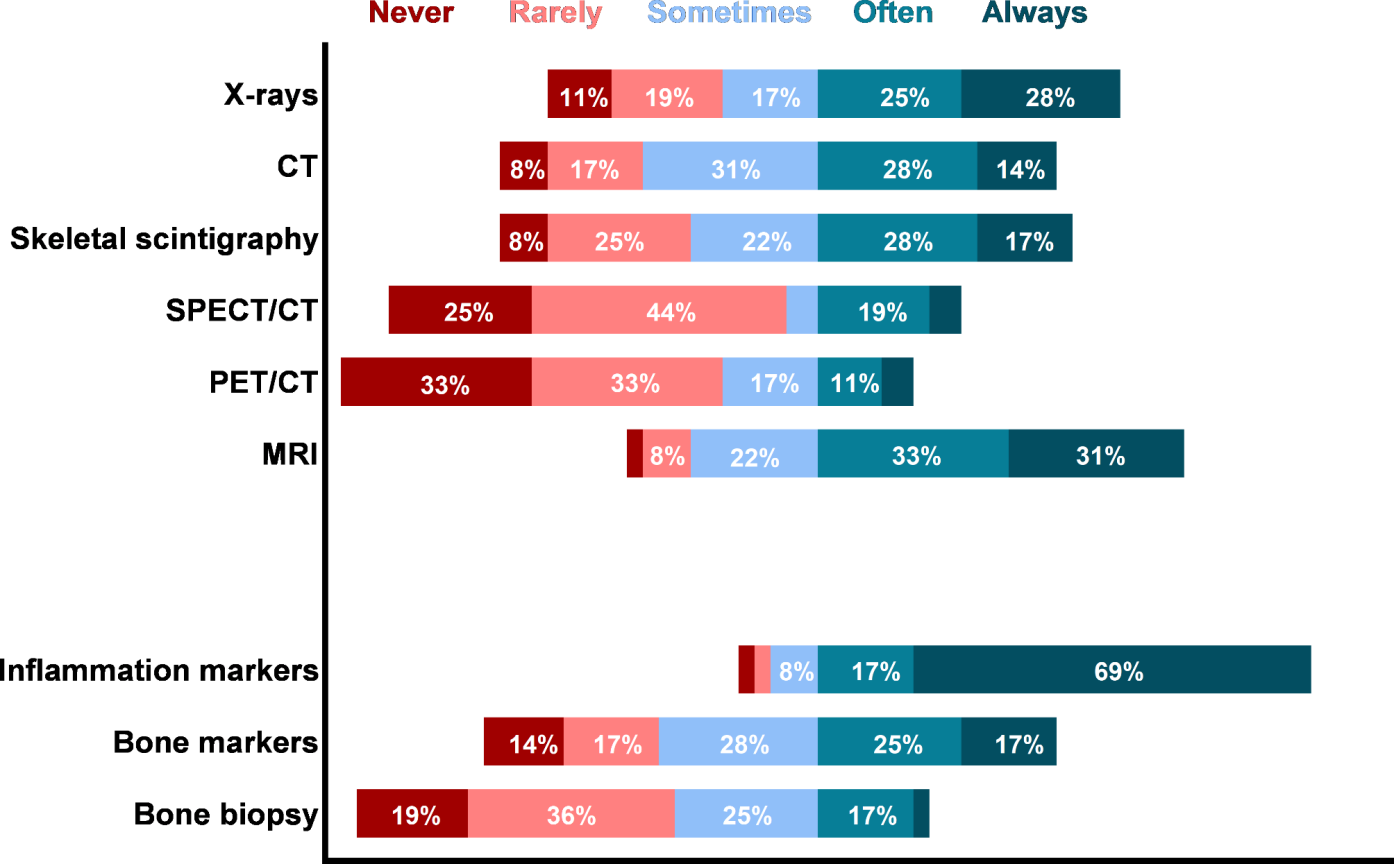
Application of diagnostic tools in adult CNO/SCCH (primary survey, n = 36). See footnote for abbreviations 3

Magnetic Resonance Imaging (MRI), X-rays and CT were frequently used to diagnose CNO/SCCH (almost/often used in 64%, 53%, 42% respectively) (Fig. 6). Bone scintigraphy Single Photon Emission Computed Tomography (SPECT) with CT, and Positron Emission Tomography (PET) with CT, were used least frequently. MRI was also favoured as imaging tool by the majority of physicians (47%), followed by combined CT and bone scintigraphy (28%) (**additional file 4**). Typical imaging findings according to responders were hyperostosis, osteitis, osteosclerosis, and bone marrow oedema, but degenerative changes, calcification of soft tissue and bony ankylosis were not (**additional file 4**). Inflammation markers were often/always used by 86% of physicians (Fig. 6) but not regarded helpful in differentiating adult CNO from other pathology, and in disease activity monitoring (**additional file 4**). Bone markers were not routinely evaluated, nor regarded helpful to diagnose/monitor CNO. Bone biopsies were infrequently performed, and regarded a very useful/essential diagnostic by only 30% of physicians.

### Treatment

**Fig. 7:**
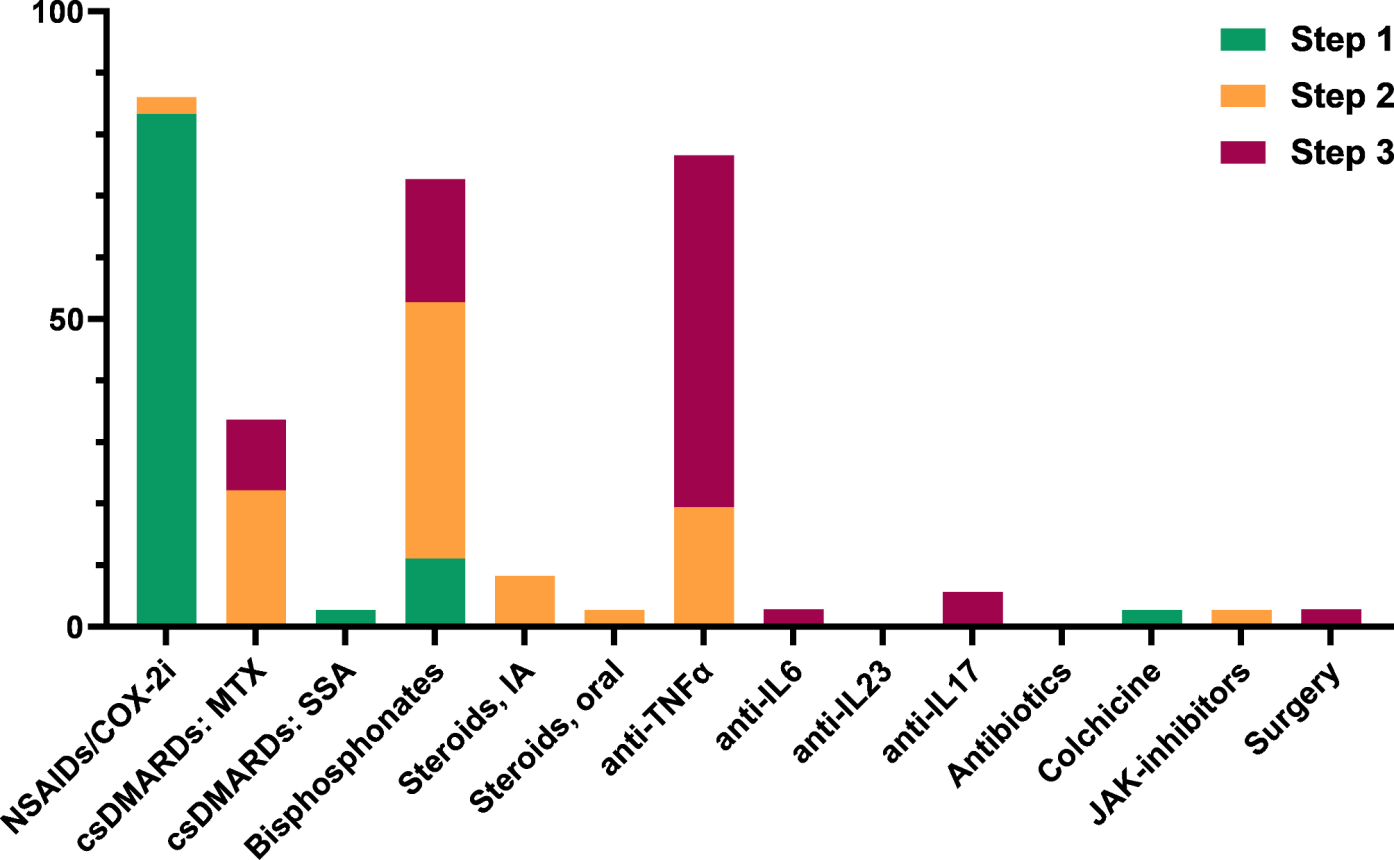
Use of step 1, step 2 and step 3 treatments in adult CNO (primary survey, n = 36). See footnote for abbreviations 4

Step 1 treatment generally consisted of non-steroidal anti-inflammatory drugs (NSAIDs) including COX-2 inhibitors (83%) (see Fig. 7). Remaining 17% of physicians installed the conventional synthetic disease modifying anti-rheumatic drug (csDMARD) sulfasalazine or colchicine, or bisphosphonates as direct step 1 treatment. Upon treatment failure, step 2–3 agents were mostly bisphosphonates, methotrexate, steroids (either intra-articular or systemic) and tumour necrosis factor alpha inhibitors (anti-TNFα). Other biologic therapy, including interleukin (IL)-6, IL-17, IL-23, and janus kinase (JAK) inhibitors were adopted by some physicians too. Antibiotics were not used by any of the responders. Within the class of bisphosphonates, intravenous pamidronate was most common, but dosage as well as regimen varied, the latter from monthly to 6-month intervals. csDMARDS and biologics were prescribed in dosages extrapolated from other rheumatic musculoskeletal diseases. Physicians, overall, considered anti-TNFα (selected by 42%) and pamidronate (selected by 39%) as most effective treatment, whichwas not affected by level of confidence in treating CNO/SCCH. As for treatment indications, presence of pain only was sufficient to initiate treatment for 26%, while for another 22% active inflammation on (nuclear) imaging or objectified swelling, redness, or warmth at physical examination was required (**see additional file 5**).

All treatment choices were primarily based on literature and expert opinion (**additional file 5**), but csDMARDs and anti-TNFα were specifically considered for their efficacy in addressing both bone inflammation and skin/joint inflammation. Determining response to treatment, physicians routinely assess patient-reported pain and functioning (96%), conduct physical exams to check for inflammatory signs (83%), and follow-up on generic inflammation parameters (61%)(see **additional file 5**). 52% and 22% of physicians reported to use follow-up MRI and bone scintigraphy respectively to track radiological and/or scintigraphic course. As ancillary treatments, 70% of physicians reported to address the importance of smoking cessation, 61% recommend physiotherapy, 17% advise on dietary interventions. The definition of remission in adult CNO included absence of inflammatory bone pain (selected by 100%), a normalization of swelling, redness and warmth at physical examination (68%), absence of biochemical inflammation (59%) and absence of radiologic inflammation (52%) (assessed by MRI (58%) or bone scintigraphy (42%) (**see additional file 5**).

## Discussion

The present study is, to our knowledge, the first to internationally map the diagnostic and therapeutic landscape of the rare disease adult CNO/SCCH. Despite the growing recognition of the CNO(/SCCH)-spectrum over time, the diagnosis and management of this condition remain fraught with challenges. In absence of validated diagnostic criteria and evidence-based therapeutic approaches, clinical care is variable and collaborative research efforts are hindered. The present study aimed to broadly delineate the current clinical practice, which may serve as input future consensus clinical guideline. Initially, our survey primarily focused on CNO/SCCH, which is a distinct subtype categorized in the ORPHA classification. This subtype specifically denotes the characteristic involvement of the anterior chest wall in adult CNO. However, our findings revealed that the usage of this name was primarily limited to the geographical regions of The Netherlands, Belgium, and Germany. Furthermore, the collected data indicated that physicians did not endorse the exclusive emphasis on CNO of the anterior chest wall in adults. Instead, they perceived the condition as part of a broader auto-inflammatory spectrum, best encapsulated by the concept of “adults with sterile bone inflammation”, and hypothetically best named “adult CNO”. Adult CNO, as observed by our responders, can also encompass a wide variety of extra-osseous features, which may invite the use of the name “SAPHO” syndrome. These insights highlight the need to adopt a more comprehensive perspective when addressing adult CNO, considering its diverse manifestations beyond the anterior chest wall involvement. Henceforth, the condition of interest will be referred to as “adult CNO” rather than “adult CNO/SCCH”.

Regardless of nomenclature, our data underscore the critical importance of establishing a standardized disease definition for adult CNO. The survey data revealed a low adherence to proposed diagnostic criteria (**see additional file 6**) highlighting subtle variations in disease definitions. As for the adherence to diagnostic criteria, we speculate that the current criteria sets are insufficiently known, and insufficiently tailored for adult CNO. Those for SAPHO are broad and can potentially include patients without bone inflammation too, which is a *sine qua non* feature for adult CNO. Alternatively, the diagnostic criteria for CNO/CRMO were originally developed based on paediatric cohorts, where differential diagnoses and clinical presentations differ. As a result, these criteria may not be well-suited for adults with the condition [10, 11, 15]. As for the subtle variations in disease definition, we found that the perceived relevance of clinical features for making diagnosis of CNO was assessed heterogeneously by responders. While relapse-remitting bone pain was positively scored (median 4.0), other features like chronic nature of complaints, absence of trauma or presence of swelling/redness at physical examination were rated so diversely that they yielded neutral summarizing scores. Second, the observed prevalence of (extra-skeletal) co-manifestations deviated among physicians, with the exception of some consistently recognized features (e.g. PPP and psoriasis; sometimes/often scored by 83% and 78% respectively). While it remains unknown though whether these differences are a result of diagnostic variation or “true clinical”, caused by environmental or genetic factors, these findings minimally suggest that cohorts across the world may be clinically distinct. For a rare disease spectrum like adult CNO, this variation could frustrate research efforts, which emphasizes the need for a standardized disease demarcation. Considering diagnostics, nuclear imaging was a relatively infrequent imaging diagnostic among physicians (bone scintigraphic SPECT with CT and PET with CT never or rarely performed by 69% and 66% respectively. This seems to contrast previous studies describing CT and nuclear imaging as ubiquitous imaging tools in adult CNO [13, 16, 17], and increased isotope uptake being a sensitive feature of the condition [9]. Responding physicians favoured MRI for diagnosis (47%). MRI has long been the recommended imaging tool in paediatric CNO, as it sensitively detects early signs of inflammation, and importantly, avoids radiation exposure [6, 18]. Why MRI also appears preferred in adult CNO cannot be fully deduced from our data, but it may be favoured owing to its ability to track inflammation more adequately than CT and bone scintigraphy. Typical findings on MRI – like bone marrow oedema - may resolve after treatment [19], while the consequences of inflammation detected by CT (sclerosis, hyperostosis) usually accumulate over time and are non-reversible (although one case was published showing full resolution of these changes after treatment [20]). Similarly, bone scintigraphy continues to demonstrate increased bone lesion isotope uptake despite clinical improvement, indicating an “imprinting pattern” [21]. In this way, MRI may theoretically be a superior disease monitoring instrument. However, in technical sense, MRI is technically challenging. Scanning time is long, study quality is varied, and risk of artefacts is high, especially in the anterior chest wall [22, 23]. These disadvantages should be considered in future discussions on the preferred diagnostic and monitoring tool for CNO. Current literature extensively describes radiological features of adult CNO [4, 24–27]. Apart from the core features of osteitis, hyperostosis, sclerosis and bone marrow oedema, adult CNO is also characterized by calcification of ligaments, ankylosis, erosions and degenerative changes like narrowing of the articular space [4, 27]. Interestingly, these latter features were not associated with diagnosis by our survey population. This may either be due to our study including just two radiologists, and thereby lacking expertise on lesser-known radiologic features. Other explanations may lie in the fact that these changes tend to occur in long-existing disease rather than in newly presenting patients [28], and in the fact these changes may be non-specific in the context of the differential diagnosis of adult CNO. In our survey, physicians indicated that inflammatory and bone turnover markers were unhelpful in diagnosing and monitoring adult CNO. Indeed, both parameters are often not elevated in the first place, making it hard to detect decreases post-treatment [9]. This illustrates how physicians are still ill-served when it comes to measuring disease activity in adult CNO. The search for sensitive, easy-to-use and specific biomarkers that adequately reflect the disease process therefore forms a major research priority. First-line treatment consisted of NSAIDs/COX-2i for practically all physicians (the two radiologists responding on common practice in their centre), but second- and third-step choices were more diverse. Resembling therapeutic practice in paediatric CNO [29], bisphosphonates, MTX and anti-TNFα were the most common second-line agents. Bisphosphonates appear to be used more in adults than in children. Other, less common, step 2 and 3 treatments reported were anti-IL17, anti-IL23, JAK inhibitors, and surgery, all of which are supported by scarce, but growing evidence of their efficacy in this condition [30–33]. Antibiotics, which were historically used upon the hypothesis that Propionibacterium Acnes was the driver the bone inflammation in CNO, were no longer used by physicians [34]. For MTX and anti-TNFα, an important treatment motivation was the convenience of targeting not only bone, but also skin and joint inflammation. In that sense, it may be rational to stratify therapy for isolated CNO (affecting bone only) and CNO plus joint and skin inflammation (more likely referred to as SAPHO). The present survey did not extensively inquire about other treatment considerations for specific patient subgroups. However, insights can be derived from the available data to conceive potential treatment approaches for these subgroups. For example, it may be reasonable to consider intra-articular steroids as a treatment option for highly localized, articular disease, while opting for potent systemic anti-inflammatory agents to target extensive multifocal bone lesions and skin inflammation. Other potential stratified approaches could involve antiresorptive therapy for patients at risk of pathological fractures, surgical management for those with localized hyperostotic complications or ankylosis, and the use of anti-IL-17 or -23 agents in individuals with pronounced psoriasis. To establish optimal patient-specific treatment approaches, further research is necessary, especially considering the clinical heterogeneity within the disease spectrum. Follow-up measures and the definition of clinical remission deviated substantially among physicians. Responders unanimously agreed that the definition of clinical remission should include the absence of pain, but 59% and 57% of physicians also required normalisation of biochemical and radiological inflammation respectively. The latter represents an important clinical discussion: as active bone inflammation is known to predispose for irreversible tissue damage and associated disease burden in the long-term, it might be premature to declare disease remission if radiologic inflammation persists [7]. Limitations of the present study include the low number of responding physicians, and the risk of sampling-bias. Still, survey responders represented a sufficiently broad set of countries and centres, providing insightful overview of current clinical practice. There was a relative lack of orthopaedic and endocrinology specialists among respondents too, but this might as well indicate that most CNO patients are cared for by rheumatologists. Still, future survey studies targeting other care providers for adult CNO (including dermatologists) may give additional insights in the disease spectrum. Another limitation of the survey was the initial focus on CNO of the anterior chest wall. While this was a deliberately choice – CNO/SCCH is a common CNO presentation in adults (affecting 89%) and often referred to as a distinct clinical entity [9] – the survey data revealed that physicians did not support this specific focus and rather perceive the disease of interest as a broad spectrum. In conclusion, adult CNO appears to be a condition embedded within a broad and insufficiently characterized disease spectrum including extra-osseous features which invite the label of SAPHO syndrome. This survey has mapped the current diagnostic and therapeutic practices in adult CNO, which are diverse and also lack in rationale as CNO pathogenesis remains largely unknown. We think our results call for an initiative to advance a clear disease spectrum definition and evidence-based treatments in the form of a first clinical guideline [5], and also to lay out a future research agenda.

## Electronic supplementary material

Below is the link to the electronic supplementary material.

Supplementary Material 1

Supplementary Material 2

Supplementary Material 3

Supplementary Material 4

Supplementary Material 5

Supplementary Material 6

Supplementary Material 7

Supplementary Material 8

Supplementary Material 9

Supplementary Material 10

Supplementary Material 11

Supplementary Material 12

## Data Availability

The datasets used and/or analysed during the current study are available from the corresponding author on reasonable request.

## List of abbreviations

Anti-TNFα: Tumor necrosis factor alpha inhibition/inhibitor
axSpA: Axial spondyloarhtritis
CNO: Chronic non-bacterial osteomyelitis
CRMO: Chronic recurrent multifocal osteomyelitis
CRP: C-reactive protein
CT: Computed tomography
CTX: C-terminal telopeptide
DMARDs: Disease modifying anti-rheumatic drugs
ESR: Erythrocyte sedimentation rate
-i: -inhibition/inhibitor
IL: Interleukin
MRI: Magnetic resonance imaging
NSAIDs: Non-steroidal anti-inflammatory drugs
PAO: Pustulotic arthro-osteitis
PE: Physical examination
PET/CT: Positron emission tomography
PPP: Pustulosis palmoplantaris
PsA: Psoriatic arthritis
P1NP: Procollagen-N-terminal-peptide
SAPHO: Synovitis, acne, pustulosis, hyperostosis, osteitis
SCC: Sternocostoclavicular
SCCH: Sternocostoclavicular hyperostosis
SPECT/CT: Single photon emission computed tomography

## Footnotes

1 *SAPHO; synovitis acne pustulosis hyperostosis osteitis syndrome, CNO; chronic nonbacterial osteomyelitis, CRMO; chronic recurrent multifocal osteomyelitis, SCCH; sternocostoclavicular hyperostosis, PAO; pustulotic arthro-osteitis*

2 *PPP; pustulosis palmoplantaris, ESR; erythrocyte sedimentation rate, CRP; C-reactive protein, AP; alkaline phosphatase, P1NP; procollagen-N-terminal-peptide, CTx; C-terminal telopeptide*

3 *CT; computed tomography, SPECT/CT; bone scintigraphic single photon emission tomography with CT, PET/CT; positron emission tomography with CT, MRI; magnetic resonance imaging, Inflammation markers; erythrocyte sedimentation rate/c-reactive protein, Bone markers; alkaline phosphatase, procollagen-N-terminal-peptide (P1NP), C-terminal telopeptide (CTX)*.

4 *NSAIDs/COX-2i; non-steroidal anti-inflammatory drugs/cyclooxygenase-2 inhibitors, csDMARDs; conventional synthetic disease modifying anti-rheumatic drugs, MTX; methotrexate, IA; intra-articular*, anti-TNFα; *tumor necrosis factor alpha inhibition, IL; interleukin-inhibition, JAK; janus kinase*
