# Supplementary material for: Diagnostic and therapeutic practices in adult chronic nonbacterial osteomyelitis (CNO)
Source: Orphanet J Rare Dis. 2023 Jul 21;18:206. doi: 10.1186/s13023-023-02831-1 (PMC10362746; doi:10.1186/s13023-023-02831-1)
Supplement: Supplementary file 1 — Supplementary Material 1 [file 13023_2023_2831_MOESM1_ESM.docx]

# Additional file 1

# Chronic nonbacterial osteomyelitis (CNO) of the sternocostoclavicular region: perspectives on diagnosis and treatment

## Primary survey

### Introductory text

Dear colleague,

We kindly invite you to take part in a **digital** **survey** on the diagnosis and treatment of chronic nonbacterial osteomyelitis (CNO) of the sternocostoclavicular region in **adults**.

CNO is a rare inflammatory bone disease spectrum. The subtype that is localized in the sternum, clavicles, and upper ribs is descriptively referred to as sternocostoclavicular hyperostosis (SCCH) and mainly occurs in adults. In this survey, we indicate this subtype as **CNO/SCCH, defined as aseptic osteitis, with sclerosis and hyperostosis of the anterior chest wall**. Adult CNO/SCCH is poorly defined. We want to align existing knowledge on adult CNO/SCCH to facilitate scientific and clinical progress.

We invite health care professionals who treat CNO/SCCH to complete two surveys: **a primary and a secondary**. This primary survey (21 questions) broadly covers current practice on classification, diagnostics and treatment in CNO/SCCH. A future secondary survey will cover these topics in more depth.

We would be thankful if you could complete this primary survey. This will take approximately 10 minutes.

We gladly receive feedback on this project. Questions can be addressed to [bot@lumc.nl](mailto:bot@lumc.nl).

Yours sincerely,

On behalf of the Center for Bone Quality, Leiden University Medical Center, Leiden, the Netherlands,

Anne Leerling, PhD Candidate
Elizabeth Winter, MD-PhD, Internist-endocrinologist

### General questions

1. Do you take care of patients with CNO/SCCH?
   - Yes
   - No

*If “no” is checked at question 1: “You indicated to not care for patients with CNO/SCCH. You will be directed to the end of the survey.”*

1. Do you give consent for the use of your personal information to contact you in the future?
   - No
   - Yes
     If so, please fill out:
     Email address:
     Country of residence: (non-obligatory)
     Institution: (non-obligatory)
2. Please indicate your profession:
   - Endocrinologist (in training or specialist)
   - Rheumatologist (in training or specialist)
   - Orthopedic surgeon (in training or specialist)
   - Endocrinology nurse practitioner
   - Rheumatology nurse practitioner
   - Orthopedic nurse practitioner
   - Other
     1. Specify:
        __________
3. Years of expertise:
   - 0-5
   - 5-10
   - 10-15
   - >15 years
4. What applies to your center? Check all that apply.
   - Academic medical center / university medical center
   - Non-academic medical center
   - Private clinic
   - Other
     1. Specify:
        __________
5. How many suspected CNO/SCCH patients are approximately referred to your center per year?
   ____ patients
6. How many diagnosed CNO/SCCH patients do you care for in your clinical practice? Choose the number that approximates your caseload best.
   - 1 per year
   - 1 per month
   - 1 per week
   - 1 per day
7. Indicate the level of confidence you feel with **diagnosing** CNO/SCCH:
   - Low
   - Little
   - Moderate
   - Fair
   - Strong
8. Indicate the level of confidence you feel with **treating** CNO/SCCH:
   - Low
   - Little
   - Moderate
   - Fair
   - Strong

### Diagnostic classification

1. When you diagnose adult CNO/SCCH (here defined as aseptic osteitis, with sclerosis and hyperostosis of the anterior chest wall), which name(s) do you use? Check all that apply.
   - Synovitis, Acne, Pustulosis, Hyperostosis, Osteitis (SAPHO) syndrome
   - Chronic nonbacterial osteomyelitis (CNO)
   - Chronic recurrent multifocal osteomyelitis (CRMO)
   - Sternocostoclavicular hyperostosis (SCCH)
   - Pustulotic arthro-osteitis/osteopathy (PAO)
   - Other
     1. Specify:
        _____________

*In case “SAPHO” is checked for Q10:*

1. When diagnosing as SAPHO syndrome, do you adhere to published diagnostic criteria? (See ancillary text for criteria list)
   - Yes, by Benhamou et al. (2003)
   - Yes, by Kahn et al. (2003, updated)
   - No

*In case “CNO or CRMO” is checked for Q10:*

1. When diagnosing as CNO or CRMO, do you adhere to published diagnostic criteria? (See ancillary text for criteria list)
   - Yes, by Jansson
   - Yes, Bristol criteria
   - No

In case neither “SAPHO”, “CNO” or “CRMO” is checked for Q10, OR when question 11/12 are checked at “no”:

1. According to you, which criteria should be met to diagnose CNO/SCCH? Please describe your diagnostic criteria in your own words:
   ___________________________________________________________________________

### Question on diagnostic tools

1. Which imaging tools do you use in diagnosing CNO/SCCH?
2. X-ray
   - Never
   - Rarely
   - Sometimes
   - Often
   - Always
3. Computed tomography (CT)
   - Never
   - Rarely
   - Sometimes
   - Often
   - Always
4. Skeletal scintigraphy
   - Never
   - Rarely
   - Sometimes
   - Often
   - Always
5. Combined CT and skeletal scintigraphy (SPECT/CT)
   - Never
   - Rarely
   - Sometimes
   - Often
   - Always
6. Positron Emission Tomography (PET)/CT scan
   - Never
   - Rarely
   - Sometimes
   - Often
   - Always
     1. If “sometimes, often or always”, please specify which radiopharmacon is used:
        ___________________________
7. Magnetic resonance imaging (MRI)
   - Never
   - Rarely
   - Sometimes
   - Often
   - Always
8. Which imaging modality is, according to your experience, most useful to diagnose adult CNO/SCCH? Choose the one which you think to have most diagnostic utility.
   - X-ray
   - CT
   - Skeletal scintigraphy
   - A combination of CT nuclear imaging (either skeletal scintigraphy (SPECT/CT) or PET/CT)
   - MRI
9. In the diagnostic work-up of CNO/SCCH, how often do you evaluate:
10. Generic inflammation markers (e.g. ESR/CRP)?
    - Never
    - Rarely
    - Sometimes
    - Often
    - Always
11. Bone markers (e.g. alkaline phosphatase, P1NP and CTx)?
    - Never
    - Rarely
    - Sometimes
    - Often
    - Always
12. Bone biopsy?
    - Never
    - Rarely
    - Sometimes
    - Often
    - Always

### Questions on imaging and clinical findings

1. What do you regard typical imaging findings indicative of CNO/SCCH?
   - Osteolytic lesions
   - Osteosclerosis
   - Hyperostosis
   - Erosion
   - (secondary) degenerative changes
   - Osteitis
   - Osteomyelitis
   - Ankylosis
   - Calcification of ligaments or soft tissue
   - Bone marrow edema
   - Other
     1. Specify:
        ______________________

### Questions on treatment of adult CNO/SCCH

1. Generally, what is your first-line treatment for adult CNO/SCCH?
   Note: following questions will cover second-line treatments, which might be administered whenever there is insufficient response to first-line treatments.
   - NSAIDs or cox-2-inhibitors
   - DMARDs: methotrexate
   - DMARDs: sulfasalazine
   - Steroids (oral)
   - Steroids (injection)
   - Bisphosphonates: pamidronate
   - Bisphosphonates: other
   - Biologicals: TNF-α inhibitor
   - Biologicals: IL-6 inhibitor
   - Biologicals: IL-23 inhibitor
   - Biologicals: IL-17 inhibitors
   - Antibiotics
   - Other
     1. Specify:
        ________________________
2. Generally, what is your second-line treatment for adult CNO/SCCH if your first-line treatment fails?
   - NSAIDs or cox-2-inhibitors
   - DMARDs: methotrexate
   - DMARDs: sulfasalazine
   - Steroids (oral)
   - Steroids (injection)
   - Bisphosphonates: pamidronate
   - Bisphosphonates: other
   - Biologicals: TNF-α inhibitor
   - Biologicals: IL-6 inhibitor
   - Biologicals: IL-23 inhibitor
   - Biologicals: IL-17 inhibitors
   - Antibiotics
   - Other
     1. Specify:
        ________________________
3. Generally, what is your third-line treatment for adult CNO/SCCH if your second-line treatment fails?
   - NSAIDs or cox-2-inhibitors
   - DMARDs: methotrexate
   - DMARDs: sulfasalazine
   - Steroids (oral)
   - Steroids (injection)
   - Bisphosphonates: pamidronate
   - Bisphosphonates: other
   - Biologicals: TNF-α inhibitor
   - Biologicals: IL-6 inhibitor
   - Biologicals: IL-23 inhibitor
   - Biologicals: IL-17 inhibitors
   - Antibiotics
   - Other
     1. Specify:
        ________________________
4. In your clinical experience, which treatment yields the best results?
   - NSAIDs or cox-2-inhibitors
   - DMARDs: methotrexate
   - DMARDs: sulfasalazine
   - Steroids (oral)
   - Steroids (injection)
   - Bisphosphonates: pamidronate
   - Bisphosphonates: other
   - Biologicals: TNF-α inhibitor
   - Biologicals: IL-6 inhibitor
   - Biologicals: IL-23 inhibitor
   - Biologicals: IL-17 inhibitors
   - Antibiotics
   - Other
     1. Specify:
        ________________________

## Secondary survey

### Introductory text

Dear colleague,

Recently you have completed a digital primary survey on the diagnosis and treatment of chronic nonbacterial osteomyelitis (CNO) of the sternocostoclavicular region in **adults**. Thank you for your contribution!

This secondary survey contains 19 questions and addresses the themes from the primary survey in more depth. We would be thankful if you could complete these questions, which will take approximately 10 minutes.

The goal of this project is to align existing knowledge on adult CNO/SCCH to facilitate scientific and clinical progress.

We gladly receive feedback on this project. Questions can be addressed to [bot@lumc.nl](mailto:bot@lumc.nl).

Yours sincerely,

On behalf of the Center for Bone Quality, Leiden University Medical Center, Leiden, the Netherlands,

Anne Leerling, PhD Candidate
Elizabeth Winter, MD-PhD, Internist-endocrinologist

### Clinical presentation

1. In your experience, how often do patients with CNO/SCCH, on top of the sterncostoclavicular manifestations, have the following other (extra)-skeletal manifestations?
2. Osteitis of the spine:
   - Never
   - Rarely
   - Sometimes
   - Often
   - Always
3. Osteitis of the jaw:
   - Never
   - Rarely
   - Sometimes
   - Often
   - Always
4. Osteitis in the peripheral skeleton:
   - Never
   - Rarely
   - Sometimes
   - Often
   - Always
5. Axial arthritis:
   - Never
   - Rarely
   - Sometimes
   - Often
   - Always
6. Peripheral arthritis:
   - Never
   - Rarely
   - Sometimes
   - Often
   - Always
7. Pustulosis palmoplantaris:
   - Never
   - Rarely
   - Sometimes
   - Often
   - Always
8. Psoriasis:
   - Never
   - Rarely
   - Sometimes
   - Often
   - Always
9. Acne:
   - Never
   - Rarely
   - Sometimes
   - Often
   - Always
10. Hidradenitis suppurativa:
    - Never
    - Rarely
    - Sometimes
    - Often
    - Always
11. Other
    - Please specify:
      ________________
    - Never
    - Rarely
    - Sometimes
    - Often
    - Always

### Diagnostic tools

*In the primary survey, you have indicated to ____ determine generic inflammation markers when diagnosing CNO/SCCH.*

1. How utile do you find generic inflammation markers (ESR/CRP) in discriminating between CNO/SCCH and other differential diagnoses?
   - Not at all useful
   - A little useful
   - Very useful
   - Essential
2. How utile do you find generic inflammation markers (ESR/CRP) in reflecting disease activity in CNO/SCCH?
   - Not at all useful
   - A little useful
   - Very useful
   - Essential

*In the primary survey, you have indicated to ____ determine bone markers when diagnosing CNO/SCCH.*

1. How utile do you find bone markers (alkaline phosphatase, P1NP and CTx) in discriminating between CNO/SCCH and other differential diagnoses?
   - Not at all useful
   - A little useful
   - Very useful
   - Essential
2. How utile do you find bone markers (alkaline phosphatase, P1NP and CTx) in reflecting disease activity in CNO/SCCH?
   - Not at all useful
   - A little useful
   - Very useful
   - Essential

*In the primary survey, you have indicated to ____ perform a bone biopsy when diagnosing CNO/SCCH.*

1. How utile do you find a bone biopsy in diagnosing CNO/SCCH?
   - Not at all useful
   - A little useful
   - Very useful
   - Essential

### Prioritizing diagnostic criteria

*In the primary survey we have asked broadly what criteria should be met for the diagnosis of CNO/SCCH, as a criteria set for this CNO subtype is lacking.*

1. Please indicate for the following criteria how essential you regard them for the diagnosis of CNO/SCCH*.*

*Imaging criteria*

- - - 1. Osteitis/osteomyelitis of the clavicles, upper ribs or sternum as proven by imaging
         - Irrelevant
         - A bit relevant
         - Relevant but not essential
         - Quite essential
         - Indispensable (i.e. diagnosis cannot be made without this criterium)
      2. Osteosclerosis of the clavicles, upper ribs or sternum as proven by imaging
         - Irrelevant
         - A bit relevant
         - Relevant but not essential
         - Quite essential
         - Indispensable (i.e. diagnosis cannot be made without this criterium)
      3. Osteolysis of the clavicles, upper ribs or sternum as proven by imaging
         - Irrelevant
         - A bit relevant
         - Relevant but not essential
         - Quite essential
         - Indispensable (i.e. diagnosis cannot be made without this criterium)
      4. Hyperostosis of the clavicles, upper ribs or sternum as proven by imaging
         - Irrelevant
         - A bit relevant
         - Relevant but not essential
         - Quite essential
         - Indispensable (i.e. diagnosis cannot be made without this criterium)
      5. Strongly increased bone metabolism as reflected by increased isotope uptake on nuclear imaging at lesional sites
         - Irrelevant
         - A bit relevant
         - Relevant but not essential
         - Quite essential
         - Indispensable (i.e. diagnosis cannot be made without this criterium)

*Clinical criteria*

- - - 1. (Relapse-remitting) bone pain at lesional sites
         - Irrelevant
         - A bit relevant
         - Relevant but not essential
         - Quite essential
         - Indispensable (i.e. diagnosis cannot be made without this criterium)
      2. Swelling (bony and/or soft tissue) at lesional sites
         - Irrelevant
         - A bit relevant
         - Relevant but not essential
         - Quite essential
         - Indispensable (i.e. diagnosis cannot be made without this criterium)
      3. Chronic nature (> 6 months course)
         - Irrelevant
         - A bit relevant
         - Relevant but not essential
         - Quite essential
         - Indispensable (i.e. diagnosis cannot be made without this criterium)
      4. Absence of trauma
         - Irrelevant
         - A bit relevant
         - Relevant but not essential
         - Quite essential
         - Indispensable (i.e. diagnosis cannot be made without this criterium)
      5. Clinical improvement on anti-inflammatory therapy
         - Irrelevant
         - A bit relevant
         - Relevant but not essential
         - Quite essential
         - Indispensable (i.e. diagnosis cannot be made without this criterium)
      6. Clinical improvement on anti-resorptive therapy
         - Irrelevant
         - A bit relevant
         - Relevant but not essential
         - Quite essential
         - Indispensable (i.e. diagnosis cannot be made without this criterium)

*Histological criteria*

- - - 1. Absence of pathogens (sterile inflammation) as proven by bone biopsy
         - Irrelevant
         - A bit relevant
         - Relevant but not essential
         - Quite essential
         - Indispensable (i.e. diagnosis cannot be made without this criterium)
      2. Absence of malignancy as proven by bone biopsy
         - Irrelevant
         - A bit relevant
         - Relevant but not essential
         - Quite essential
         - Indispensable (i.e. diagnosis cannot be made without this criterium)

*Biochemical criteria*

- - - 1. Generic inflammatory markers mildly to moderately elevated
         - Irrelevant
         - A bit relevant
         - Relevant but not essential
         - Quite essential
         - Indispensable (i.e. diagnosis cannot be made without this criterium)

### Treatment: initiation

1. What contributes to your decision to start treatment for adult CNO/SCCH? Check all that apply.
   - Inflammatory bone pain at lesional site
   - Any type of pain (e.g. mechanical, neuropathic) at lesional site
   - Functional impairment at or near lesional site
   - Active inflammation on (nuclear) imaging
   - Elevated inflammatory markers in laboratory investigation
   - Local swelling, warmth, or redness at lesional site
   - The diagnosis of CNO/SCCH itself, regardless of symptomatology
2. Which of the following should minimally be present to start treatment for CNO/SCCH (indication)? Check all that apply.
   - Inflammatory bone pain at lesional site
   - Any type of pain (e.g. mechanical, neuropathic) at lesional site
   - Functional impairment at or near lesional site
   - Active inflammation on (nuclear) imaging
   - Elevated inflammatory markers in laboratory investigation
   - Local swelling, warmth, or redness at lesional site
   - No specific indication: any CNO/SCCH has indication for treatment, with or without symptoms
3. What are your treatment goals in CNO/SCCH? Check all that apply.
   - Absence of pain
   - Decrease of pain
   - Absence of functional impairment (patient-reported)
   - Decrease of functional impairment (patient-reported)
   - Free range of motion (objectified)
   - Improved range of motion (objectified)
   - Normalisation of radiologic inflammation
   - Decrease of radiologic inflammation
   - Normalisation of bone turnover on nuclear imaging
   - Decrease of bone turnover on nuclear imaging
   - Stabilisation of structural changes of bone and surrounding tissue
   - Prevention of complications
   - Other
     1. Please specify:
        _________________________

### Treatment contents and monitoring

*In the primary survey, you indicated that “___” was your first-line treatment for CNO/SCCH.*

1. Why is this your first-line treatment for CNO/SCCH? Check all reasons that apply.
   - Based on literature
   - Expert opinion
   - Patient’s preference
   - Convenience (e.g. targets multiple manifestations; bone and skin inflammation)
   - Other
     1. Specify:
        ________________
2. Specify the type (name), dosage, frequency of administration and duration for your first-line treatment:
   ______________________
3. After how many months do you determine response to treatment?
   ___ months
4. How do you determine response to treatment? Check all that contribute to this assessment.
   - Patient-reported pain and functioning
   - Follow up imaging: X-ray
   - Follow up imaging: CT
   - Follow up imaging: Skeletal scintigraphy
   - Follow up imaging: combination of CT nuclear imaging (either skeletal scintigraphy (SPECT/CT) or PET/CT)
   - Follow up imaging: MRI
   - Laboratory investigation: inflammation markers (CRP/ESR)
   - Laboratory investigation: markers of bone turnover (alkaline phosphatase, P1NP and CTx)
   - Physical examination
   - Other:
     1. Specify:
        ___________________________________

*In the primary survey, you indicated that “___” was your second-line treatment for CNO/SCCH.*

1. Why is this your second-line treatment for CNO/SCCH? Check all reasons that apply.
   - Based on literature
   - Expert opinion
   - Patient’s preference
   - Convenience (e.g. targets multiple manifestations; bone and skin inflammation)
   - Other
     1. Specify:
        ________________
2. Specify the type (name), dosage, frequency of administration and duration for your second-line treatment:
   ______________________
3. After how many months do you determine response to treatment?
   ___ months
4. How do you determine response to treatment? Check all that contribute to this assessment.
   - Patient-reported pain and functioning
   - Follow up imaging: X-ray
   - Follow up imaging: CT
   - Follow up imaging: Skeletal scintigraphy
   - Follow up imaging: combination of CT nuclear imaging (either skeletal scintigraphy (SPECT/CT) or PET/CT)
   - Follow up imaging: MRI
   - Laboratory investigation: inflammation markers (CRP/ESR)
   - Laboratory investigation: markers of bone turnover (alkaline phosphatase, P1NP and CTx)
   - Physical examination
   - Other:
     1. Specify:
        ___________________________________

*In the primary survey, you indicated that “___” was your third-line treatment for CNO/SCCH.*

1. Why is this your third-line treatment for CNO/SCCH? Check all reasons that apply.
   - Based on literature
   - Expert opinion
   - Patient’s preference
   - Convenience (e.g. targets multiple manifestations; bone and skin inflammation)
   - Other
     1. Specify:
        ________________
2. Specify the type (name), dosage, frequency of administration and duration for your third-line treatment:
   ______________________
3. After how many months do you determine response to treatment?
   ___ months
4. How do you determine response to treatment? Check all that contribute to this assessment.
   - Patient-reported pain and functioning
   - Follow up imaging: X-ray
   - Follow up imaging: CT
   - Follow up imaging: Skeletal scintigraphy
   - Follow up imaging: combination of CT nuclear imaging (either skeletal scintigraphy (SPECT/CT) or PET/CT)
   - Follow up imaging: MRI
   - Laboratory investigation: inflammation markers (CRP/ESR)
   - Laboratory investigation: markers of bone turnover (alkaline phosphatase, P1NP and CTx)
   - Physical examination
   - Other:
     1. Specify:
        ___________________________________
5. Which of the following non-pharmacological treatments do you recommend/start in patients with CNO/SCCH? Choose all that apply.
   - Physiotherapy
   - Smoking cessation
   - Dietary interventions
   - None of the above
   - Other:
     1. Specify:
        ___________________________________
6. How do you define remission of CNO/SCCH? Check those that are required in your definition of remission.
   - Absence of inflammatory bone pain at lesional sites
   - Absence of inflammation or increased bone metabolism on (nuclear) imaging
   - Normal inflammatory markers in laboratory investigation
   - Absence of local swelling, warmth, or redness at lesional site
7. Would you be interested to attend an expert consensus meeting on CNO/SCCH hosted by the European Calcified Tissue Society (ECTS) with the goal to reach consensus on unresolved issues deriving from the primary and secondary survey? If you check “yes” or “possibly”, you will receive further information by e-mail.
   - Yes
   - Possibly
   - No
