# Supplementary material for: Diagnostic and therapeutic practices in adult chronic nonbacterial osteomyelitis (CNO)
Source: Orphanet J Rare Dis. 2023 Jul 21;18:206. doi: 10.1186/s13023-023-02831-1 (PMC10362746; doi:10.1186/s13023-023-02831-1)
Supplement: Supplementary file 2 — Supplementary Material 2 [file 13023_2023_2831_MOESM2_ESM.docx]

# Additional file 2

Overview of networks approached for dissemination of primary survey

|  | Network |
| --- | --- |
| European | European Alliance of Associations for Rheumatology (EULAR) |
|  | Rare Immunodeficiency, Autoinflammatory and Autoimmune Diseases Network (ERN RITA) |
|  | European Reference Network on Connective Tissue and Musculoskeletal Diseases (ERN ReCONNET) |
|  | European Reference Network on Rare Bone Disorders (ERN BOND) |
|  | European Society of Endocrinology (ESE) |
|  | European Calcified Tissue Society (ECTS), Rare Bone Disease Action Group |
| Asia/pacific area | South East Asia and Pacific Area League Against Rheumatism (APLAR) |
|  | Japan College of Rheumatology (JCR) |
| North America | American Society for Bone and Mineral Research (ASBMR) |
| Global | International Osteoporosis Foundation (IOF) |
|  | International Federation of Musculoskeletal Research Societies (IFMRS) |
| Dutch | Nederlandse Vereniging voor Endocrinologie, Bone (NvE Bone) |
