# Supplementary material for: Diagnostic and therapeutic practices in adult chronic nonbacterial osteomyelitis (CNO)
Source: Orphanet J Rare Dis. 2023 Jul 21;18:206. doi: 10.1186/s13023-023-02831-1 (PMC10362746; doi:10.1186/s13023-023-02831-1)
Supplement: Supplementary file 4 — Supplementary Material 4 [file 13023_2023_2831_MOESM4_ESM.docx]

# Additional file 6: Proposed criteria sets for SAPHO syndrome and CNO/CRMO

**Table 1:** Proposed criteria sets for SAPHO syndrome, and CNO/CRMO (derived from pediatric cohorts)

| **SAPHO by Kahn (1994)** | **SAPHO by Kahn (2003)** | **CNO by Jansson (2009)** | **CNO/CRMO Bristol criteria (2016)** |
| --- | --- | --- | --- |
| Principal   - Aseptic multifocal osteomyelitis or caused by *prioprionibacterium acnes*   Lesser   - Osteoarticular: acute or chronic joint involvement, monostotic or polyostotic aseptic osteitis - PPP, severe acne, hidradenitis suppurativa   *Diagnosis made in presence of principal criterium or 2 lesser criteria.* | Inclusion (minimally 1)   - Bone-joint involvement associated with PPP and psoriasis vulgaris - Bone-joint involvement associated with severe acne - Isolated sterile (exempting proprionibacterium acnes) hyperostosis/osteitis (adults) - Chronic recurrent multifocal osteomyelitis (children) - Bone-joint involvement associated with chronic bowel diseases   Exclusion:   - Infectious osteitis - Tumoral conditions of the bone - Noninflammatory condensing lesions of the bone | Major:   - Radiologically proven osteolytic/-sclerotic bone lesions - Multifocal bone lesions - PPP or psoriasis - Sterile bone biopsy with signs of inflammation and/or fibrosis, sclerosis   Minor:   - Normal blood count and good general state of health - CRP and ESR mildly-to-moderately elevated - Observation time > 6 months - Hyperostosis - Associated with other autoimmune diseases apart from PPP or psoriasis - Grade I or II relatives with autoimmune or autoinflammatory disease   *Diagnosis made in presence of 2 major or 1 major and 3 minor criteria.* | - Typical clinical findings (bone pain with or without localized swelling without significant local or systemic features of inflammation or infection)   AND   - Typical radiological findings (plain x-ray: showing combination of lytic areas, sclerosis and new bone formation or preferably STIR MRI showing bone marrow edema with or without bone expansion, lytic areas and periosteal reaction.   AND EITHER   - More than one bone (or clavicle alone) without significantly raised CRP (<30 g/L) - If unifocal disease (other than clavicle) or CRP > 30 g/L with bone biopsy showing inflammatory changes (plasma cells, osteoclasts, fibrosis, or sclerosis) with no bacterial growth whilst not on antibiotic therapy |

Legend: CRP; c-reactive protein, PPP; palmoplantar pustulosis
