# Supplementary material for: Diagnostic and therapeutic practices in adult chronic nonbacterial osteomyelitis (CNO)
Source: Orphanet J Rare Dis. 2023 Jul 21;18:206. doi: 10.1186/s13023-023-02831-1 (PMC10362746; doi:10.1186/s13023-023-02831-1)
Supplement: Supplementary file 6 — Supplementary Material 6 [file 13023_2023_2831_MOESM6_ESM.docx]

# Additional file 5: Treatment initiation, goals, response measures and reported regimens

**Figure:** Motivations for treatment choices for NSAIDs, bisphosphonates, DMARDs and TNFi (secondary survey, n=23)
*Legend: NSAIDs/COX-2i; non-steroidal anti-inflammatory drugs/cyclooxygenase-2 inhibitors, DMARDs; disease modifying anti-rheumatic drugs, TNFi; tumor necrosis factor alpha inhibitor*

**Figure:** Strict indications to initiate treatment for CNO/SCCH (secondary survey, total n=23)
*Legend: PE; physical examination*

**Figure:** Treatment goals of responding physicians for CNO/SCCH (secondary survey, n=23)

**Figure:** Treatment response measured (checked by % of total, secondary survey, n=23)

**D:** Definition of remission (secondary survey, n=23)

**Additional data:** reported treatment regimens:

Most commonly used NSAID-regimen was Naproxen 500 mg 2 times daily and treatment response or failure was generally declared at 3 months.

Pamidronate intravenous (IV) regimens were diverse, including 3x30 mg or 3x60 mg every 3 months, and 1x60 mg every three months, 1 month or 2 weeks.

Other bisphosphonates which were reported were zoledronate IV, at 4-5 mg varying per month, every 3, 6 or 12 months or ibandronate 3x3mg IV monthly. Bisphosphonate response was mostly assessed at 4-6 months.

For csDMARDs (methotrexate and to lesser extent sulfasalazine) and anti-TNFα (etanercept, adalimumab, infliximab) were mostly administered in regular rheumatological regimens, and response mostly determined at 3 months for both.
