# Supplementary material for: Diagnostic and therapeutic practices in adult chronic nonbacterial osteomyelitis (CNO)
Source: Orphanet J Rare Dis. 2023 Jul 21;18:206. doi: 10.1186/s13023-023-02831-1 (PMC10362746; doi:10.1186/s13023-023-02831-1)
Supplement: Supplementary file 8 — Supplementary Material 8 [file 13023_2023_2831_MOESM8_ESM.docx]

**Additional file 3:**

Additional clinical features as observed in adult CNO/SCCH:

- Uveitis (n=3, all “rarely”)
- Dactylitis (n=1, “rarely”)
- Arthritis mutilans with erosive arthritis (n=1, “rarely”)
- Inflammatory bowel disease (n=1, “rarely”)
- Enthesitis (n=1, “often”)
- Depression (n=1, “often”)
- History of malignancy (n=1, “often”)
- Poor dental status (n=1, “often”)
- Takayasu arthritis (n=1, “rarely”)

Categorized responses on the diagnostic definition of adult CNO, according to responders.

| Response category | # Responses Total n=25* |
| --- | --- |
| Typical imaging findings (alone) *Imaging findings reported: sterile osteitis, hyperostosis, sclerosis, hypercaptation on nuclear imaging, bone marrow edema, osteolysis, synovitis of the sternoclavicular joint* | N=7 |
| Typical histological findings (alone):  *Histological findings reported: sterile osteitis, osteolysis, osteosclerosis, benign aspect* | N=3 |
| Typical imaging + clinical findings (combined) *Clinical findings reported: pain, movement restriction of adjacent joints, joint swelling, low to mildly elevated inflammation markers, response to anti-inflammatory therapy, association with (neutrophilic) dermatoses* | N=7 |
| Typical imaging + clinical findings + exclusion of differential diagnoses *Differential diagnoses to be excluded: infectious osteomyelitis, other rheumatic diseases like axial spondylarthritis (axSpA)* | N=3 |
| Typical imaging + clinical findings + pustulosis palmoplantaris (PPP) | N=1 |
| Diagnostic criteria are not useful in clinical practice | N=2 |
| Diagnosing as axial spondylarthritis (axSpA) or psoriatic arthritis (PsA) | N=1 |
| Do not know | N=1 |

*Remaining 11 physicians indicated to use existing criteria sets, see figure 3 of main manuscript
