# Supplementary material for: Diagnostic and therapeutic practices in adult chronic nonbacterial osteomyelitis (CNO)
Source: Orphanet J Rare Dis. 2023 Jul 21;18:206. doi: 10.1186/s13023-023-02831-1 (PMC10362746; doi:10.1186/s13023-023-02831-1)
Supplement: Supplementary file 11 — Supplementary Material 11 [file 13023_2023_2831_MOESM11_ESM.docx]

# Additional file 4: Imaging preferences and features and utility of diagnostic tools

**Figure:** preferred diagnostic imaging tool as rated by physicians (n=36)

**Figure:** Physicians’ perspectives regarding typical imaging features of adult CNO/SCCH (primary survey, n=36)

**Figure:** Utility of diagnostic tools in differentiating between adult CNO/SCCH and other diagnoses, and in disease activity monitoring (secondary survey, n=23)
*Legend: ESR; erythrocyte sedimentation rate, CRP; C-reactive protein, bone markers; alkaline phosphatase, procollagen-N-terminal-peptide (P1NP), C-terminal telopeptide (CTX)*
