# Supplementary material for: Diagnostic and therapeutic practices in adult chronic nonbacterial osteomyelitis (CNO)
Source: Orphanet J Rare Dis. 2023 Jul 21;18:206. doi: 10.1186/s13023-023-02831-1 (PMC10362746; doi:10.1186/s13023-023-02831-1)
Supplement: Supplementary file 12 — Supplementary Material 12 [file 13023_2023_2831_MOESM12_ESM.docx]

**Listed response to reviewer comments**
Submission OJRD-D-23-00117

Dear Editor,

Herewith we are pleased to submit our revised manuscript entitled “Diagnostic and therapeutic practices in adult chronic nonbacterial osteomyelitis (CNO)” (original submission OJRD-D-23-00117).

We have adjusted the manuscript according to the concerns of the reviewers. In our view, the raised issues have been adequately addressed.

Please find below a listed response to the reviewer comments, referring to the relevant revised sections.

Yours sincerely,

On behalf of all authors,

Anne T. Leerling

**Comments reviewer 1:**
In this study, the authors reported an international landscape to understand the diagnostic and therapeutic practices in adult chronic nonbacterial osteomyelitis/Synovitis Acne Pustulosis Hyperostosis Osteitis syndrome (CNO/SAPHO).

Major Points:

Please write detailed information of Chronic nonbacterial osteomyelitis (CNO) in the "Introduction" part of the manuscript.

We added a more comprehensive introduction of CNO, including clinical presentation and an overview of the disease spectrum and its subtypes. Section now reads:

**Lines 102-123:** “Chronic nonbacterial osteomyelitis (CNO, ORPHA: 324964) is a rare disease spectrum marked by auto-inflammatory bone lesions and locally elevated bone turnover. Patients may present with inflammatory bone pain, swelling and functional impairment, which profoundly impacts quality of life (1, 2). Over time, persistent bone inflammation may lead to irreversible structural tissue damage, characterized by sclerosis, erosions, new bone formation, and degenerative changes, emphasizing the need for timely diagnosis and intervention (3, 4). Despite the increasing awareness for CNO in the past decades, clinical management remains challenging due to the absence of evidence-based treatments (5).	CNO demonstrates a broad clinical phenotype, encompassing both paediatric and adult presentations. In children, CNO is characterized by multiple alternating bone lesions that mostly affect the long bones and is usually referred to as chronic recurrent multifocal osteomyelitis (CRMO, shared ORPHA code with CNO: 324964). Adult CNO comprises different subtypes, including sternocostoclavicular hyperostosis (SCCH, ORPHA: 178311), indicating specific involvement of the anterior chest wall, and Synovitis, Acne, Pustulosis, Hyperostosis, Osteitis (SAPHO) syndrome, indicating more systemic involvement with joint and skin pathology (ORPHA: 793). Adults may also display a multifocal disease pattern that resembles paediatric CRMO (6-9).	At present, it is unknown to what degree the adult CNO-spectrum shares a common genetic or immunological base and should be addressed as a coherent entity, or whether subtypes differ in pathophysiology warranting different therapeutic approach. In any case, there are no validated classification criteria for the adult CNO spectrum as a whole, nor for its (presumed) subtypes. Proposed criteria for the CNO-spectrum were either derived from paediatric populations, or aim to frame the broad and heterogeneous entity of SAPHO syndrome (10-12).”

Please write detailed information of Characteristics of respondents in the "Result" part of the manuscript.

We added more detailed data of responders. Section now reads:

**Lines 175-184:** “48 physicians completed the primary survey, of whom 36, mainly rheumatologists, represented the target population of care providers for adult CNO/SCCH patients (figure 1). Of these, 23 completed the secondary survey. Responses mainly derived from Europe (64%), and also from North America (11%), South America (3%), Asia (6%) and Africa (3%) (figure 2A). Physicians were mostly based at university medical centres (86%), non-university medical centres (19%) and several practiced at private clinics (8%). Most physicians had >10 or 5-10 years of clinical experience (78% and 14% respectively). Median number of patients referred for suspected CNO/SCCH was 5 per year (IQR 2-14). 64% stated to see 1-5 confirmed patients annually, 22% see 1-5 patients per month, and 14% see 1-5 patients per week. Physicians were mostly strongly or fairly confident in diagnosing and treating adult CNO/SCCH (83% and 72% respectively) (figure 2B).”

Please describe the criteria for Nomenclature.

We appreciate that the reviewer points this out. In fact, it was an objective of our study to investigate which nomenclature physicians use in adults with sterile bone inflammation, considering the existence of multiple names used for this condition. Our survey revealed that more than half of the physicians use different names for the condition depending on patient profile, rather than using one name for the full spectrum. For instance, they may refer to the condition as "CNO" when only bone inflammation is observed, or use the term "SAPHO" when dermatologic or articular symptoms are also present. None of these labels are supported by validated diagnostic criteria, emphasizing the need of harmonization. The majority of physicians did not adhere to the criteria sets proposed in literature in their clinical practice. We have added these nuances in the results section, and also address this theme more extensively in the discussion. Sections now read:

**Lines 191-201, results:** “Given the variety of labels used to indicate the patient population of CNO/SCCH, the survey inquired about the use of these labels in clinical practice. 50% of physicians used one term consistently to describe adult CNO/SCCH, whereas 50% used multiple terms depending on the clinical picture. SAPHO was used most frequently (78%) (figure 3). CNO and CRMO were used by 36% and 33% respectively and SCCH was used by 31% (solely by physicians from The Netherlands, Belgium and Germany). Among physicians favouring ‘SAPHO’ and/or ‘CNO/CRMO’ as terms, a minority indicated to adhere to the proposed criteria sets proposed for these labels, that is the sets of Kahn 1994/2003 for SAPHO, and the sets deriving from paediatric studies for CNO/CRMO (Bristol 2016 or Jansson 2007) (10-12, 15). Pustulotic arthro-osteitis (PAO), a term that derives from dermatology and indicates bone inflammation in the context of pustulosis palmoplantaris (PPP), was used by a single physician.”

**Lines 312-333, discussion:** “Initially, our survey primarily focused on CNO/SCCH, which is a distinct subtype categorized in the ORPHA classification. This subtype specifically denotes the characteristic involvement of the anterior chest wall in adult CNO. However, our findings revealed that the usage of this name was primarily limited to the geographical regions of The Netherlands, Belgium, and Germany. Furthermore, the collected data indicated that physicians did not endorse the exclusive emphasis on CNO of the anterior chest wall in adults. Instead, they perceived the condition as part of a broader auto-inflammatory spectrum, best encapsulated by the concept of "adults with sterile bone inflammation", and hypothetically best named “adult CNO”. Adult CNO, as observed by our responders, can also encompass a wide variety of extra-osseous features, which may invite the use of the name “SAPHO” syndrome. These insights highlight the need to adopt a more comprehensive perspective when addressing adult CNO, considering its diverse manifestations beyond the anterior chest wall involvement. Henceforth, the condition of interest will be referred to as “adult CNO” rather than “adult CNO/SCCH”.	Regardless of nomenclature, our data underscore the critical importance of establishing a standardized disease definition for adult CNO. The survey data revealed a low adherence to proposed diagnostic criteria (see additional file 6) highlighting subtle variations in disease definitions. As for the adherence to diagnostic criteria, we speculate that the current criteria sets are insufficiently known, and insufficiently tailored for adult CNO. Those for SAPHO are broad and can potentially include patients without bone inflammation too, which is a *sine qua non* feature for adult CNO. Alternatively, the diagnostic criteria for CNO/CRMO were originally developed based on paediatric cohorts, where differential diagnoses and clinical presentations differ. As a result, these criteria may not be well-suited for adults with the condition (10, 11, 13).”

In this study, please describe the clinical presentation & diagnostic features.

The *frequency* of clinical features in adult CNO/SCCH as observed by physicians is discussed in lines 210-217 and shown in figure 4. Subsequently, free-text disease definitions of adult CNO/SCCH are discussed with reference to additional file 3, followed by the *relevance* of certain diagnostic features to diagnose CNO/SCCH in adults (as depicted in figure 5). We have revised this section to further clarify these results, which now read:

**Lines 210-239:** “The presence of clinical features as observed in adult CNO/SCCH (i.e. in adults with sterile bone inflammation of the anterior chest wall) is depicted in figure 4. Generally, many features are seen in “some” cases, reflecting a heterogeneous clinical presentation. Physicians reported different frequencies of osteitis of the jaw, peripheral arthritis, and hidradenitis suppurativa; these were scored occurring as “never” (13%, 13%, 17%) almost as frequently as “often” (9%, 17%, 9%). Most consistently rated as prevalent features were PPP, followed by psoriasis, axial arthritis and peripheral and spinal osteitis. Other rare features that (single) physicians reported in an open field question are listed in additional file 3.	To gain a deeper understanding of the physician's conceptualization of CNO/SCCH, the survey included a follow-up question for physicians who indicated to *not* use diagnostic criteria sets to identify their patients (n=25) (additional file 3). A free-text diagnostic definition of adult CNO/SCCH was asked. Following categorization of the responses, the definition seemed to rely mostly on imaging. Imaging features alone (n=7) or in conjunction with distinctive clinical features (n=7) were leading in diagnosing adult CNO/SCCH. A minority of physicians (n=3) emphasized the necessity of histological evidence to confirm the presence of sterile bone inflammation. Furthermore, 3 physicians indicated that diagnosis of adult CNO/SCCH requires the exclusion of other diagnoses such as axial spondylarthritis. Importantly, the majority of responses did not incorporate the anterior chest wall as a specifically typical localization. Instead, responses embodied the broader concept of "sterile bone inflammation, with or without accompanying extra-skeletal inflammatory manifestations". Building on the free-text input of physicians and use of existing criteria sets, physicians were asked to score the *individual* *relevance* of specific features to diagnose adult CNO/SCCH (figure 5). Most positively rated were radiologically proven osteitis/osteomyelitis (median (IQR) 4.0 (4.0-4.0)), relapse-remitting bone pain, sclerosis, hyperostosis, and increased uptake on nuclear imaging (all 4.0 (3.0-4.0)). Shoulder movement restriction and biochemical parameters were generally rated less or irrelevant. The remainder of features were rated mostly neutral, as relevant but not essential for diagnosis. “

Please list the diagnostic tools you used in this study.

The diagnostic tools physician use in their clinical practice are shown in **figure 6**. In addition, **lines 249-260** and **additional file 4** refer to this topic.

Please illustrate and discuss the treatment and therapeutics in details.

As requested, we added a more detailed discussion on treatment in the result section, partly with reference to **additional file 5** and in the discussion of the manuscript. Sections now read:

**Lines 268-282, results:** “Step 1 treatment generally consisted of non-steroidal anti-inflammatory drugs (NSAIDs) including COX-2 inhibitors (83%) (see figure 7). Remaining 17% of physicians installed the conventional synthetic disease modifying anti-rheumatic drug (csDMARD) sulfasalazine or colchicine, or bisphosphonates as direct step 1 treatment. Upon treatment failure, step 2-3 agents were mostly bisphosphonates, methotrexate, steroids (either intra-articular or systemic) and tumour necrosis factor alpha inhibitors (anti-TNFα). Other biologic therapy, including interleukin (IL)-6, IL-17, IL-23, and janus kinase (JAK) inhibitors were adopted by some physicians too. Antibiotics were not used by any of the responders. Within the class of bisphosphonates, intravenous pamidronate was most common, but dosage as well as regimen varied, the latter from monthly to 6-month intervals. csDMARDS and biologics were prescribed in dosages extrapolated from other rheumatic musculoskeletal diseases. Physicians, overall, considered anti-TNFα (selected by 42%) and pamidronate (selected by 39%) as most effective treatment, which was not affected by level of confidence in treating CNO/SCCH. As for treatment indications, presence of pain only was sufficient to initiate treatment for 26%, while for another 22% active inflammation on (nuclear) imaging or objectified swelling, redness, or warmth at physical examination was required (see additional file 5).”

**Lines 289-302, results:** “All treatment choices were primarily based on literature and expert opinion (additional file 5), but csDMARDs and anti-TNFα were specifically considered for their efficacy in addressing both bone inflammation and skin/joint inflammation. Determining response to treatment, physicians routinely assess patient-reported pain and functioning (96%), conduct physical exams to check for inflammatory signs (83%), and follow-up on generic inflammation parameters (61%)(see additional file 5). 52% and 22% of physicians reported to use follow-up MRI and bone scintigraphy respectively to track radiological and/or scintigraphic course. As ancillary treatments, 70% of physicians reported to address the importance of smoking cessation, 61% recommend physiotherapy, 17% advise on dietary interventions. The definition of remission in adult CNO included absence of inflammatory bone pain (selected by 100%), a normalization of swelling, redness and warmth at physical examination (68%), absence of biochemical inflammation (59%) and absence of radiologic inflammation (52%) (assessed by MRI (58%) or bone scintigraphy (42%) (see additional file 5).”

**Lines 386-407, discussion:** “First-line treatment consisted of NSAIDs/COX-2i for practically all physicians (the two radiologists responding on common practice in their centre), but second- and third-step choices were more diverse. Resembling therapeutic practice in paediatric CNO (14), bisphosphonates, MTX and anti-TNFα were the most common second-line agents. Bisphosphonates appear to be used more in adults than in children. Other, less common, step 2 and 3 treatments reported were anti-IL17, anti-IL23, JAK inhibitors, and surgery, all of which are supported by scarce, but growing evidence of their efficacy in this condition (15-18). Antibiotics, which were historically used upon the hypothesis that Propionibacterium Acnes was the driver the bone inflammation in CNO, were no longer used by physicians (19).	For MTX and anti-TNFα, an important treatment motivation was the convenience of targeting not only bone, but also skin and joint inflammation. In that sense, it may be rational to stratify therapy for isolated CNO (affecting bone only) and CNO plus joint and skin inflammation (more likely referred to as SAPHO). The present survey did not extensively inquire about other treatment considerations for specific patient subgroups. However, insights can be derived from the available data to conceive potential treatment approaches for these subgroups. For example, it may be reasonable to consider intra-articular steroids as a treatment option for highly localized, articular disease, while opting for potent systemic anti-inflammatory agents to target extensive multifocal bone lesions and skin inflammation. Other potential stratified approaches could involve antiresorptive therapy for patients at risk of pathological fractures, surgical management for those with localized hyperostotic complications or ankylosis, and the use of anti-IL-17 or -23 agents in individuals with pronounced psoriasis. To establish optimal patient-specific treatment approaches, further research is necessary, especially considering the clinical heterogeneity within the disease spectrum.”

There are some typos and grammar mistakes in the text and need some editorial improvement.

Please, delete irrelevant "general" phrases and sentences.

The paper was thoroughly revised for language and style. Generalities were deleted as recommended by the reviewer.

Overall written English need to improve.

The paper was reviewed by the native speaking co-author Dr. Gavin Clunie.

**Comments reviewer 2:**
The authors reported diagnostic and therapeutic practices for CNO using two surveys. They aimed to form a general view based on expert opinions. There are major points to consider.

The most significant flaw of this study is the low number of respondents. The authors could revise the title since the current version implies a high number of international participants.

We agree with the reviewer that the sample size of the present study is low, which may be a direct effect of the rarity and scattered care as discussed in the limitations of the study in lines 417-427. We have adapted the title on the reviewer’s request, which now reads: **“Diagnostic and therapeutic practices in adult chronic nonbacterial osteomyelitis (CNO)”.**

The second significant flaw was that the invitation was written as CNO/sternoclavicular joint involvement, but the title is CNO/SAPHO, the article seemed generally about the CNO approaches, not specifically sternoclavicular joint involvement.

We thank the reviewer for this comment as it concerns a complex and vital aspect of the discussion concerning the spectrum of this autoinflammatory bone disease. Indeed, the initial focus of the survey was adults with sterile bone inflammation of the anterior chest wall (CNO/SCCH). However, the survey data revealed that responding physicians do not perceive CNO/SCCH as a distinct subtype to the same extent as we had anticipated. Instead, they reported a highly diverse and broad spectrum of clinical presentations, and seemed to have completed the questions with the broader definition of “the adult with sterile bone inflammation” in mind. We have included this newfound insight more extensively in the results section and also open the discussion with this highly important finding and its implications. We have adapted the title, which now refers to “adult chronic nonbacterial osteomyelitis” in general, to convey the width of the responses received. Revised sections now read:

**Lines 223-235, results:** “To gain a deeper understanding of the physician's conceptualization of CNO/SCCH, the survey included a follow-up question for physicians who indicated to *not* use diagnostic criteria sets to identify their patients (n=25) (additional file 3). A free-text diagnostic definition of adult CNO/SCCH was asked. Following categorization of the responses, the definition seemed to rely mostly on imaging. Imaging features alone (n=7) or in conjunction with distinctive clinical features (n=7) were leading in diagnosing adult CNO/SCCH. A minority of physicians (n=3) emphasized the necessity of histological evidence to confirm the presence of sterile bone inflammation. Furthermore, 3 physicians indicated that diagnosis of adult CNO/SCCH requires the exclusion of other diagnoses such as axial spondylarthritis. Importantly, the majority of responses did not incorporate the anterior chest wall as a specifically typical localization. Instead, responses embodied the broader concept of "sterile bone inflammation, with or without accompanying extra-skeletal inflammatory manifestations". Building on the free-text input of physicians and use of existing criteria sets, physicians were asked to score the *individual* *relevance* of specific features to diagnose adult CNO/SCCH (figure 5).”

**Lines 312-324, discussion:** “Initially, our survey primarily focused on CNO/SCCH, which is a distinct subtype categorized in the ORPHA classification. This subtype specifically denotes the characteristic involvement of the anterior chest wall in adult CNO. However, our findings revealed that the usage of this name was primarily limited to the geographical regions of The Netherlands, Belgium, and Germany. Furthermore, the collected data indicated that physicians did not endorse the exclusive emphasis on CNO of the anterior chest wall in adults. Instead, they perceived the condition as part of a broader auto-inflammatory spectrum, best encapsulated by the concept of "adults with sterile bone inflammation", and hypothetically best named “adult CNO”. Adult CNO, as observed by our responders, can also encompass a wide variety of extra-osseous features, which may invite the use of the name “SAPHO” syndrome. These insights highlight the need to adopt a more comprehensive perspective when addressing adult CNO, considering its diverse manifestations beyond the anterior chest wall involvement. Henceforth, the condition of interest will be referred to as “adult CNO” rather than “adult CNO/SCCH”.”

Methods: EU survey tool should be explained in more detail. In the statistical analysis part, the methods used to analyze the normality of the distribution in numeric variables should be mentioned.

We have added, as requested, details on the survey-management system of EUSurvey and statistic methods of numerical data in the method section.

These sections now read:

**Lines 137-138:** “The surveys were facilitated by the software of EUSurvey, an online survey-management system (20).”

**Lines 165-166:** “Numerical data are presented as mean ± standard deviation (SD) or median (IQR) for normal and non-normal data, as assessed by graphical inspection and Shapiro-Wilk test.”

Results: The authors could explore further the results regarding the indication and specific type of biologic drugs or pamidronate and the response (regarding skin and bone disease) to treatment (if these were addressed in the questionnaire).

We thank the reviewer for this suggestion. However, the questionnaire did not address this into detail. However, we did add a more detailed discussion on therapeutic management in the result section and figure 7, partly with reference to additional file 5. The discussion now also includes considerations regarding therapeutic stratification (lines 386-407). The sections now read:

**Lines 268-282, results:** “Step 1 treatment generally consisted of non-steroidal anti-inflammatory drugs (NSAIDs) including COX-2 inhibitors (83%) (see figure 7). Remaining 17% of physicians installed the conventional synthetic disease modifying anti-rheumatic drug (csDMARD) sulfasalazine or colchicine, or bisphosphonates as direct step 1 treatment. Upon treatment failure, step 2-3 agents were mostly bisphosphonates, methotrexate, steroids (either intra-articular or systemic) and tumour necrosis factor alpha inhibitors (anti-TNFα). Other biologic therapy, including interleukin (IL)-6, IL-17, IL-23, and janus kinase (JAK) inhibitors were adopted by some physicians too. Antibiotics were not used by any of the responders. Within the class of bisphosphonates, intravenous pamidronate was most common, but dosage as well as regimen varied, the latter from monthly to 6-month intervals. csDMARDS and biologics were prescribed in dosages extrapolated from other rheumatic musculoskeletal diseases. Physicians, overall, considered anti-TNFα (selected by 42%) and pamidronate (selected by 39%) as most effective treatment, which was not affected by level of confidence in treating CNO/SCCH. As for treatment indications, presence of pain only was sufficient to initiate treatment for 26%, while for another 22% active inflammation on (nuclear) imaging or objectified swelling, redness, or warmth at physical examination was required (see additional file 5).”

**Lines 289-302, results:** “All treatment choices were primarily based on literature and expert opinion (additional file 5), but csDMARDs and anti-TNFα were specifically considered for their efficacy in addressing both bone inflammation and skin/joint inflammation. Determining response to treatment, physicians routinely assess patient-reported pain and functioning (96%), conduct physical exams to check for inflammatory signs (83%), and follow-up on generic inflammation parameters (61%)(see additional file 5). 52% and 22% of physicians reported to use follow-up MRI and bone scintigraphy respectively to track radiological and/or scintigraphic course. As ancillary treatments, 70% of physicians reported to address the importance of smoking cessation, 61% recommend physiotherapy, 17% advise on dietary interventions. The definition of remission in adult CNO included absence of inflammatory bone pain (selected by 100%), a normalization of swelling, redness and warmth at physical examination (68%), absence of biochemical inflammation (59%) and absence of radiologic inflammation (52%) (assessed by MRI (58%) or bone scintigraphy (42%) (see additional file 5).”

**Lines 386-407, discussion:** “First-line treatment consisted of NSAIDs/COX-2i for practically all physicians (the two radiologists responding on common practice in their centre), but second- and third-step choices were more diverse. Resembling therapeutic practice in paediatric CNO (14), bisphosphonates, MTX and anti-TNFα were the most common second-line agents. Bisphosphonates appear to be used more in adults than in children. Other, less common, step 2 and 3 treatments reported were anti-IL17, anti-IL23, JAK inhibitors, and surgery, all of which are supported by scarce, but growing evidence of their efficacy in this condition (15-18). Antibiotics, which were historically used upon the hypothesis that Propionibacterium Acnes was the driver the bone inflammation in CNO, were no longer used by physicians (19).	For MTX and anti-TNFα, an important treatment motivation was the convenience of targeting not only bone, but also skin and joint inflammation. In that sense, it may be rational to stratify therapy for isolated CNO (affecting bone only) and CNO plus joint and skin inflammation (more likely referred to as SAPHO). The present survey did not extensively inquire about other treatment considerations for specific patient subgroups. However, insights can be derived from the available data to conceive potential treatment approaches for these subgroups. For example, it may be reasonable to consider intra-articular steroids as a treatment option for highly localized, articular disease, while opting for potent systemic anti-inflammatory agents to target extensive multifocal bone lesions and skin inflammation. Other potential stratified approaches could involve antiresorptive therapy for patients at risk of pathological fractures, surgical management for those with localized hyperostotic complications or ankylosis, and the use of anti-IL-17 or -23 agents in individuals with pronounced psoriasis. To establish optimal patient-specific treatment approaches, further research is necessary, especially considering the clinical heterogeneity within the disease spectrum.”

Clinical presentation & diagnostic features paragraph: Takayasu and malignancy in the open field questions, could be explained in more detail.

These clinical co-manifestations of adult CNO/SCCH were provided by a single physician in an optional free-text field. We have made the decision to relocate them (and other n=1 responses) to additional file 3.

Discussion: 'Despite increased isotope uptake being rated so positively as a diagnostic indicator, bone scintigraphy was a relatively unpopular imaging diagnostic among physicians (never or rarely performed by 69% (Bone scintigraphic SPECT with CT) and 66% (PET with CT) respectively). This contrasts with previous studies proposing CT and nuclear imaging as useful imaging tools in adult CNO/SAPHO'. Being 'useful' and 'unpopular' are expressed as opposites but their meanings are different. It might be good to phrase it differently.

Rephrased accordingly in lines 350-354, which now read: “Considering diagnostics, nuclear imaging was a relatively infrequent imaging diagnostic among physicians (bone scintigraphic SPECT with CT and PET with CT never or rarely performed by 69% and 66% respectively). This seems to contrast previous studies describing CT and nuclear imaging as ubiquitous imaging tools in adult CNO (21-23), and increased isotope uptake being a sensitive feature of the condition (9).”

There are several typos in the text, and the text could be carefully edited (for instance page 8 line 195 could be Takayasu arteritis instead of Takayasu arthritis).

The paper was thoroughly revised for language, style and coherency.

Abbreviations in figure legends could be written in the footnotes.

The abbreviations in the figure legends were moved to the footnotes.

**Comments by reviewer 3:**
Very informative article on CNO and SAPHO and the derivation of the different definitions of adult CNO and SAPHO according to the representative survey of experts. Extensive and detailed description of the results with meaningful illustrations.

In the Discussion, it could be added that there is a history in the therapy of SAPHO/CNO: Antibiotics formerly in use, now no longer.

Following the reviewer’s suggestion, we have added this nuance in lines 392-394, which read: “Antibiotics, which were historically used upon the hypothesis that Propionibacterium Acnes was the driver the bone inflammation in CNO, were no longer used by physicians (19).”

Similarly, scintigraphy used to be most important diagnostic tool now rather MRI. It is also interesting to note that the importance of HLAB27 is essentially no longer relevant, for the future, however, genetic research could be relevant. Perhaps it could be discussed in more detail whether dermatologists and pediatricians should be included in a questionnaire survey to see how these specialist groups view the focus of the CNO /SAPHO.

We agree with the reviewer's suggestion that conducting an additional survey targeting dermatologists and pediatricians could provide valuable insights. It is noteworthy, however, that pediatricians have already been surveyed regarding their diagnostic approaches and management strategies in pediatric CNO/CRMO, as referenced (14). Furthermore, an existing publication has examined the disparities in treatment approaches between dermatologists and rheumatologists (24). We have included this consideration in lines 421-422 of the discussion, which read as follows: “Still, future survey studies targeting other care providers for adult CNO (including dermatologists) may give additional insights in the disease spectrum”.

I think the article is also related to the planned meeting in June and October this year and provides an excellent basis for further discussions to achieve a clearer definition of patients with CNO/SAPHO, not least to enable therapies in defined patient groups for the first time in clinical trials.
We thank the reviewer for this comment.
